# Exploiting the Potential of *Drosophila* Models in Lysosomal Storage Disorders: Pathological Mechanisms and Drug Discovery

**DOI:** 10.3390/biomedicines9030268

**Published:** 2021-03-07

**Authors:** Laura Rigon, Concetta De Filippis, Barbara Napoli, Rosella Tomanin, Genny Orso

**Affiliations:** 1 Fondazione Istituto di Ricerca Pediatrica “Città della Speranza”, Corso Stati Uniti 4, 35127 Padova, Italy; concetta.defilippis93@gmail.com (C.D.F.); rosella.tomanin@unipd.it (R.T.); 2 Laboratory of Diagnosis and Therapy of Lysosomal Disorders, Department of Women’s and Children’s Health, University of Padova, Via Giustiniani 3, 35128 Padova, Italy; 3 Laboratory of Molecular Biology, Scientific Institute, IRCCS Eugenio Medea, Via Don Luigi Monza 20, Bosisio Parini, 23842 Lecco, Italy; barbaranapoli89@gmail.com; 4 Department of Pharmaceutical and Pharmacological Sciences, University of Padova, Via Marzolo 5, 35131 Padova, Italy; genny.orso@unipd.it

**Keywords:** lysosomal storage disorders, *Drosophila melanogaster*, animal model, mucopolysaccharidosis, sphingolipidosis, mucolipidosis, neuronal ceroid lipofuscinosis, autophagy, lysosome

## Abstract

Lysosomal storage disorders (LSDs) represent a complex and heterogeneous group of rare genetic diseases due to mutations in genes coding for lysosomal enzymes, membrane proteins or transporters. This leads to the accumulation of undegraded materials within lysosomes and a broad range of severe clinical features, often including the impairment of central nervous system (CNS). When available, enzyme replacement therapy slows the disease progression although it is not curative; also, most recombinant enzymes cannot cross the blood-brain barrier, leaving the CNS untreated. The inefficient degradative capability of the lysosomes has a negative impact on the flux through the endolysosomal and autophagic pathways; therefore, dysregulation of these pathways is increasingly emerging as a relevant disease mechanism in LSDs. In the last twenty years, different LSD *Drosophila* models have been generated, mainly for diseases presenting with neurological involvement. The fruit fly provides a large selection of tools to investigate lysosomes, autophagy and endocytic pathways in vivo, as well as to analyse neuronal and glial cells. The possibility to use *Drosophila* in drug repurposing and discovery makes it an attractive model for LSDs lacking effective therapies. Here, ee describe the major cellular pathways implicated in LSDs pathogenesis, the approaches available for their study and the *Drosophila* models developed for these diseases. Finally, we highlight a possible use of LSDs *Drosophila* models for drug screening studies.

## 1. Introduction

Lysosomal storage disorders (LSDs) are a group of about 70 inherited metabolic diseases [1] caused by deficiencies in lysosomal acid hydrolases, membrane proteins or transporters. This results in abnormal accumulation of undegraded macromolecules within the endolysosomal system and dysregulation of this pathway [2]. Although many LSDs had already been recognized as clinical entities since the 19th century, their classification started many years later with the discovery of the lysosome by Christian de Duve in 1955 [3] and with the establishment of the concept of lysosomal diseases by Hers in 1965 [4]. Typically, LSDs are primarily classified based on the biochemical nature of the storage material: sphingolipidoses, gangliosidoses, leukodystrophies, mucopolysaccharidoses, glycoproteinoses, mucolipidoses and cystinosis [5]. They are inherited in an autosomal recessive manner, except for Mucopolysaccharidosis type II, Fabry disease and Danon disease, which are X-linked. Individually rare, their prevalence varies from 7.5 to 23.5 per 100,000 live births [6]. Most LSDs present a broad phenotypic spectrum, ranging from severe forms with infantile-onset to milder ones with adult-onset. The most common clinical signs include hepatosplenomegaly, musculoskeletal deformities, pulmonary and cardiac problems, deafness, blindness and movement impairment. In addition, two-thirds of the patients present with heavy neurological involvement [5].

Since the discovery of LSDs, important research efforts have been made to find effective treatments for these pathologies. However, despite great successes and progress, many LSDs still lack a cure or can benefit from a therapy, as enzyme replacement, which is ineffective on neurological or bone symptoms. To overcome these limitations, certainly, a thorough understanding of their pathogenesis would be of great help. Thus, in recent years several studies of basic biological processes concerning endolysosomal pathway, autophagy, lysophagy and mitochondria have been conducted, allowing to better clarify the link among these pathways, together with inflammatory and developmental defects, all differently involved in LSDs [5]. However, efforts are still needed to fully understand the multiple aspects underlying lysosomal storage and what they entail and probably only a deeper comprehension of this could lead to the development of effective therapies, especially for difficult to treat areas as the central nervous system (CNS).

To understand the pathophysiology of LSDs and to develop possible therapies, vertebrate models (mainly zebrafish, mouse, cat, dog, sheep) and invertebrate models (*C. elegans*, *D. melanogaster*) have been generated since the 1970s [7,8,9,10,11]. In particular, the invertebrate models for LSDs have been developed since the beginning of the 2000s, first in worm [12] and then in fruit fly [13].

This review summarizes the major cellular pathways implicated in LSDs and the *Drosophila melanogaster* models that have been developed for these diseases, discussing the key findings that they have allowed, their potential for future studies and the importance of developing new therapies, in particular their use for drug screening.

## 2. Cellular Pathways Involved in LSDs

### 2.1. The Lysosome and Its Spatial Distribution in the Cytosol

#### 2.1.1. Lysosome Structure and Formation

**Figure 1:**
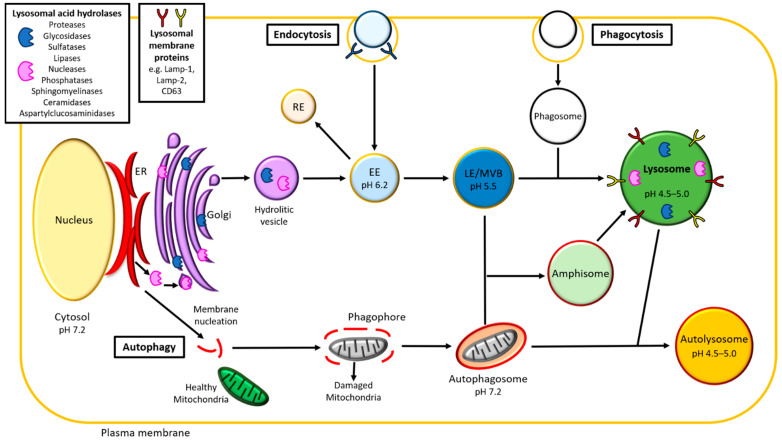
Endolysosomal and autophagic pathways. Schematic view of the main cellular pathways involved in Lysosomal Storage Diseases. As summarized by the figure, endolysosomal and autophagic pathways are the most important processes regulating the degradation and recycling of intracellular materials. These two pathways converge to the final step of lysosomes (green) formation. EE, early endosome (light blue); LE, late endosome (blue); MVB, multivesicular body (blue); RE, recycling endosome (light yellow); ER, endoplasmic reticulum (red); Golgi (violet); amphisome (light green); autolysosome (orange); healthy mitochondria (dark green); damaged mitochondria (grey).

Lysosomes are membrane-bounded organelles for a long time identified mainly as the “cell recycling center”, later recognized as the center of the cell homeostasis and in the past decade as an important component of the organelle network [14,15]. The lysosomal membrane is enriched with over 120 membrane proteins. Most of them (primarily LAMPs and LIMPs) are glycosylated in the luminal domain, forming the so-called glycocalyx, that protects the membrane from internal digestion [16,17]. The main transmembrane multimeric protein complex is the H ± ATPase, responsible for the characteristic acidic pH (4.5–5.0) of the lysosomal lumen, essential for the functioning of the acidic hydrolases. In fact, they are activated exclusively at this pH and this also prevents their action in other cellular compartments. The lysosomal lumen contains about 60 acid hydrolases (proteases, glycosidases, sulfatases, lipases, nucleases, phosphatases, sphingomyelinases, ceramidases and aspartylglucosaminidases), specialized in the degradation of intra- and extra-cellular macromolecules, such as lipids, polysaccharides, proteins and nucleic acids [18] (Figure 1).

In its primary function of “lytic body” of the cell, the lysosome actively degrades the discarded or damaged intra- and extra-cellular material, to maintain cellular homeostasis and guarantee the correct disposal of the different macromolecules and organelles as well as the correct recovery of their products for further metabolic processes and cellular functions.

Lysosome function and formation are regulated and guaranteed by two main pathways: endocytosis and autophagy (Figure 1). In particular, the endocytosis (or endolysosomal pathway) allows internalization and recycling of extracellular materials, whereas autophagy allows the degradation and turnover of organelles and large intra-cellular macromolecules.

The first step in the formation of the lysosome is the budding from the Golgi apparatus of hydrolytic vesicles (or primary lysosome), enriched in the lithic enzymes produced by the endoplasmic reticulum (ER). After synthesis in the ER, acid hydrolases are phosphorylated in the Golgi with a mannose-6-phosphate (M6P) residue, which allows the binding to the M6P receptors (MPRs) in the trans-Golgi network (TGN) and the transport to the early endosomes (EEs) and late endosomes (LEs) via clathrin-coated vesicles, joining to the endolysosomal pathway and, finally, forming the lysosomes [19].

#### 2.1.2. Lysosome Positioning

Early positioning studies of lysosomes identified a preference of these organelles for the perinuclear space [20]. However, studies conducted over the past 30 years have highlighted that lysosomes are widely distributed throughout the cytoplasm [21]. In non-polarized cells lysosomes are mainly concentrated in the perinuclear cloud surrounding the microtubule organizing center, whereas in polarized cells, such as neurons, they are distributed in all cytoplasmatic domains [5]. Kinesin and dynein motors regulate the anterograde and retrograde movement of lysosomes along microtubules, in a stop-and-go motion [21]. Contact with others organelles can also influence lysosome motility; for example, endoplasmic reticulum (ER)-lysosome contact leads lysosomes to localize to the perinuclear area, where fusion and fission of organelles take place [5]. Even if they move throughout the cell, the lysosomes maintain non-random and well-defined spatial distributions at the whole-cell scale [22]. Moreover, the single lysosomes are able to organize together to increase their density in an area and to interact with the endosomes [22]. These abilities of moving and maintaining stable spatial distribution are the basis of the involvement of these special organelles in multiple functions.

### 2.2. Functions of the Lysosome and Its Central Role in Cell Homeostasis

#### 2.2.1. Lysosome as a Regulatory Hub

In the last years, the study of lysosome positioning and its capacity to fuse with other intracellular membranes and organelles contributed to elucidate the many functions played by lysosomes, in addition to being the cell recycling center [15]. It plays a key role in processes like endocytosis, autophagy, secretion, lipid metabolism, glycogen homeostasis, plasma membrane repair, gene regulation, nutrient sensing, ion homeostasis, pathogen sensing and immune responses. These many functions have led to the definition of the lysosome as a “regulatory hub” of the cell [15,23,24]. Lysosomes are now considered not only in the study of LSDs pathogenesis, but also in other more common and studied disorders, such as neurodegenerative diseases and cancer [25].

In this review, we will summarize the role of the lysosome in key processes such as endocytosis and autophagy. The relationship between lysosomes and lipid metabolism and glycogen homeostasis will be also discussed. Information about the other processes in which lysosomes are involved can be found in other recent reviews [23,24,25].

#### 2.2.2. Endocytosis

The endolysosomal pathway (Figure 1) starts with the budding and detachment from the plasma membrane of the endocytic vesicle. The latter then fuses with the EEs, that subsequently mature into LEs and, finally, fuse with lysosomes. Endosomes and lysosomes can interact and exchange materials by two different mechanisms: the kiss-and-run and the direct fusion. In the kiss-and-run process, the lysosome transiently merges with the endosome forming a pore that allows the exchange of cargos between the two organelles (kiss), followed by their scission in order to prevent their complete fusion (run). In the direct fusion, the lysosome completely merges with the endosome forming the endo-lysosome. Cargoes destined for degradation in lysosomes are retained in intraluminal vesicles (ILVs) within EEs, which have a mild acid pH of 6.0. The small GTPase Rab5 localizes in the EEs and it is considered the master regulator of the endolysosomal system [26]. ILVs increase during maturation of EEs to LEs, thanks to successive fusion and fission cycles of membranes, during which the lipid and protein composition of the endosomes is also modified [27]. LEs are also called multivesicular bodies (MVBs), for the numerous ILVs contained and present Rab7 as specific coat component, which replaces Rab5 in the maturation process [28]. Rab5 and Rab7 are conserved in *Drosophila* and mammals, where they are both involved in the endolysosomal and autophagic pathways [29]. Both pathways and the main genes involved are conserved among eukaryotes, allowing to study complex pathways *in vivo*, even using simpler models such as the fruit fly. In this view, in a recent study, Jacomin and colleagues blocked the endolysosomal pathway at different levels (Shibire, Rab4, Rab5, Chmp1 and Rab7), showing in the fruit fly that its integrity is necessary for proper lysosome biogenesis and effective autophagy in vivo [30].

Basic metabolites degraded in lysosomes are released in the cytosol, through specific lysosomal transporters, to be reused in different cellular processes. Some macromolecules, not destined to lysosomal degradation, can return to the plasma membrane directly from the EEs, thanks to specialized recycling endosomes (REs). These are characterized by two Rab proteins (Rab4, Rab11) [31,32] and can direct the cargo not only to the plasma membrane but also to other separate cellular destinations [33].

#### 2.2.3. Autophagy

Autophagy is a highly conserved process among different species and currently it can be classified into three subtypes: microautophagy, chaperone-mediated autophagy (CMA) and macroautophagy [34,35]. In macroautophagy (hereafter called autophagy), internal organelles (e.g., mitochondria) are enclosed by fragments of membranes from the ER, forming first phagophores and then autophagosomes (Figure 1). It starts with the sequestration of soluble materials and organelles in a newly formed double membrane called phagophore. The maturation of the double membrane and the complete inclusion of the internal cargo ends in the formation of the autophagosome. This fuses with LEs and lysosomes, forming respectively the amphisome and the autolysosome and recycling their contents.

The formation of the phagophore, size and number of autophagosomes and the autophagic activity are regulated by autophagy-related gene (ATG) proteins, together with the members of the light chain 3 (LC3) family (Atg8 in the fruit fly), vascular protein sorting (VPS), Rab small GTPases and specific SNAREs. ATG proteins are about 40, but only parts of them are directly associated with autophagy; they are highly conserved across eukaryotes, including *Drosophila melanogaster* [36].

Thanks to the first identification of autophagy in the larval fruit fly [37] and the conservation of the autophagic and the endolysosomal pathways, many studies have been carried out in *Drosophila* to understand the function of many of these proteins [29,38]. For example, one of the last studies suggested a central role of Rab2 in the regulation of both pathways, by controlling the fusion processes with the LEs [39].

Together with ATGs, mTOR (mammalian target of rapamycin) is another master regulator of autophagy. In healthy condition and nutrient availability, mTOR is related to the lysosomal membrane in a peripheral location of the cell and interacts with the transcription factor EB (TFEB) to prevent its translocation to the nucleus. In a condition of starvation, lysosomes move to the perinuclear zone and the binding of mTOR with TFEB is interrupted. Thus, TFEB is translocated to the nucleus and here it binds to CLEAR (coordinated lysosomal expression and regulation) elements, inducing the transcription of genes involved in lysosomal biogenesis and autophagy [40].

#### 2.2.4. Autophagic Lysosomal Reformation (ALR)

**Figure 2:**
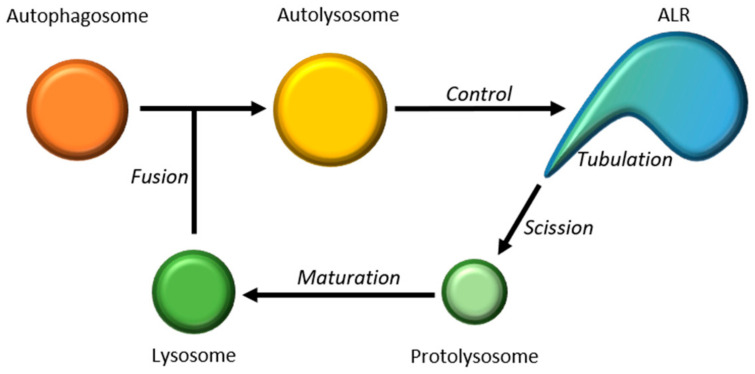
Autophagic lysosome reformation (ARL). Schematic illustration of the principal steps involved in the autophagic lysosome reformation. Initially, the tubular structures emerge from autolysosomes (yellow) and small vesicles bud off these tubules (protolysosome, in light green) to mature into functional lysosomes (dark green). The renewed pool of lysosomes can re-enter the flux fusing with the autophagosomes (orange) and generating autolysosome.

In addition to the de novo formation from the Golgi, in the last decade, another mechanism of lysosome formation has been identified and studied: the Autophagic Lysosomal Reformation (ALR) [41]. The ALR process consists in the formation of new lysosomes starting from autolysosome. This reformation starts with the clathrin-mediated membrane budding from the autolysosome, then phosphatidylinositol-4,5-biphosphate (PI4,5P2) and KIF5B trigger the elongation of the buds in Lamp1-positive membrane tubules; finally, the GTPase Dynamin 2 allows the proto-lysosome scission and the maturation to lysosomes [42] (Figure 2).

The regulation of ALR is strictly connected to the regulation of autophagy. After prolonged starvation, levels of metabolites released from the autolysosome increase and this could reactivate mTOR activity, reducing autophagy and increasing ALR process [41]. In *Drosophila*, it has been found that defects in Spinster (Spin), a lysosomal efflux permease with the function of sugar transporter, cause storage of lysosomal carbohydrates and enlarged lysosomes and autolysosomes. Thus, Spin is essential after prolonged starvation for mTOR reactivation and ALR [43].

#### 2.2.5. Glycogen Homeostasis

**Figure 3:**
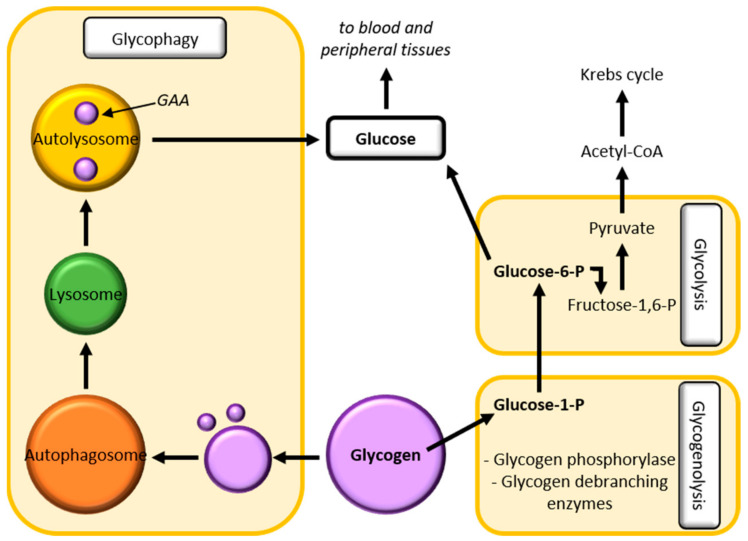
Glycogen Homeostasis. Schematic view of the main pathways involved in glycogen homeostasis: glycogenolysis, glycolysis and glycophagy. Glycogen (violet) is cleaved to glucose-1-phosphate during glycogenolysis and serves as a substrate for the glycolysis allowing the release of glucose. Glycogen homeostasis is also regulated by glycophagy in which glycogen is sequestered in lysosomes (green) and degraded to obtain glucose. Autolysosome in yellow, autophagosome in orange.

Glycogen is a highly branched polymer of glucose residues and it is present in the cytosol in the form of granules. In vertebrates, glycogen represents the storage form of the glucose and it is predominantly found in the liver and in the skeletal muscle and, in small amounts, in the brain. Three main pathways are involved in glycogen homeostasis: glycolysis, glucose release into the blood and lysosome glycogen clearance (Figure 3). The latter serves to dispose of glycogen, which arrived into lysosomes through glycophagy [44].

Glycophagy is a glycogen-specific autophagy with a key role in maintaining glucose homeostasis. It is a hormonally controlled and regulated process, in which glycogen is sequestered into lysosomes and here degraded by the lysosomal acid alpha-glucosidase (GAA), making glucose available [45]. Glucose is then released in the cytosol thanks to efflux permease and sugar transporters, like Spinster in the fruit fly [43]. Being a specific type of autophagy, glycophagy is also regulated by mTOR, which is, in turn, regulated downstream by protein phosphatase 2A (PP2A), promoting the synthesis of acid glucosidase [45]. Glycogen homeostasis could be studied in *Drosophila*, as were the endolysosomal and autophagic pathways, by which it is also regulated. Zirin and colleagues have demonstrated that glycophagy depends on autophagy genes and it is therefore also dependent on the availability of nutrients or on starvation conditions [46], suggesting that the fruit fly could be a good model to study glycophagy, as well as the other pathways involved in glycogen homeostasis.

#### 2.2.6. Lipid Metabolism

**Figure 4:**
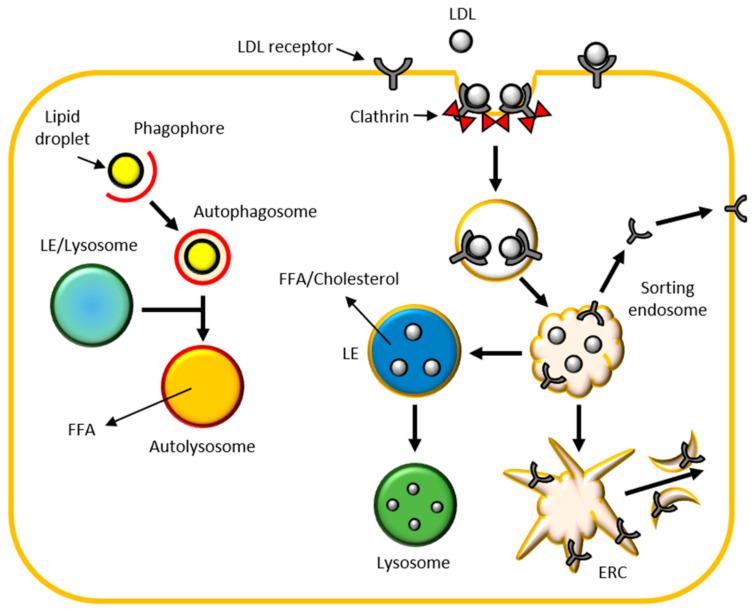
Lipid metabolism: lipid species such as triacylglycerols and sterol esters reach lysosomes (green) through different ways: as lipid bilayer of different vesicles; by endocytosis mediated by specific low-density lipoprotein (LDL) receptors; by autophagy specific for lipids (like lipid droplets), named lipophagy. LE, late endosome (blue); ERC, endocytic recycling compartment (light yellow); FFA, free fatty acid.

Lysosomes and late endosomes can degrade lipids thanks to lipases, specific acid hydrolases present in their lumen. Lipids reach lysosomes through different ways: as lipid bilayer of different vesicles; by endocytosis mediated by specific low-density lipoprotein (LDL) receptors; by autophagy specific for lipids (like lipid droplets), named lipophagy (Figure 4).

Hydrolysis of lipids in LEs and lysosomes requires highly controlled mechanisms. Indeed, lipases could also degrade the lipid bilayer of the organelles and, therefore, two systems are mainly used by lysosomes and LEs to protect themselves. First, the abundance of highly glycosylated lysosomal membrane proteins, such as the lysosomal integral membrane proteins (LIMPs) and lysosome-associated membrane proteins (LAMPs), forms the glycocalyx which is enzyme-resistant [16]. Second, lipid hydrolysis is mainly carried out in the internal vesicle membranes, that are highly enriched in BMP (bis(monoacylglycerol)phosphate), an unusual negative charged glycerophospholipid [47].

LDLs lead exogenous sterols, triglycerides and phospholipids to degradation in lysosomes [48]. LDLs bind to their specific receptors on the plasma membrane, they are internalized by clathrin-coated vesicles and transported to transient sorting endosomes. Here, LDLs are directly recycled towards the plasma membranes or passing through the endocytic recycling compartment (ERC). The sorting endosomes mature into LEs that start to degrade LDLs internalized in the ILVs and enriched in BMP. LEs mature into lysosomes and LDLs degradation proceeds releasing cholesterol and free fatty acids (FFA) (Figure 4). Cholesterol is insoluble in the aqueous environment of the lysosomal lumen. Niemann-Pick type C (NPC) 2 protein acts delivering cholesterol through the lysosomal lumen to the luminal N-terminal domain of NPC1 on the lysosomal membrane. Hence, cholesterol is released from lysosome and transported to ER, plasma membrane and Golgi through different vesicular and non-vesicular trafficking routes [49].

Lipid droplets (LDs) are lipid-rich organelles that regulate intracellular lipid storage and metabolism. In recent years, lipophagy has been considered increasingly important as a mechanism for regulating lipid homeostasis. In this autophagic process, LDs are internalized in the bilayer of the phagophore, which matures in autophagosome and then fuses with the LEs and/or lysosomes to form the autolysosomes, where the lipids are degraded to FFA [49].

Very little is known about the regulation of lipid metabolism in LSDs and for this purpose the use of *Drosophila* would be very helpful. Indeed, the fruit fly shares with mammals most of the organs and the metabolic signalling pathways involved in lipid metabolism and homeostasis. They are evolutionarily and functionally conserved and its fat body (similar to adipose tissue in mammals) is full of lipid droplets and easy to study [50].

### 2.3. Lysosomal Diseases and the Major Metabolic Pathways Altered in in These Pathologies

Dysfunctions in endolysosomal and autophagic pathways are implicated in several disorders [23]. This has led in recent years to the identification of a broader class of diseases defined lysosomal disorders, which include not only the most classic lysosomal storage diseases, but also all those pathologies that have been found to have a lysosomal impairment [15]. These also include such well-known neurodegenerative diseases such as Parkinson’s, Alzheimer’s and Huntington’s diseases.

In the classic LSDs the accumulation is mainly caused by lysosomal acid hydrolases deficit, but also by impairment of proteins involved in lysosomal trafficking (e.g., LAMP2, LIMP2, NPC1, NPC2) [1]. The different degradation cascades are compromised, as well as the consequent availability of metabolites and the cellular homeostasis. Given the role of a metabolic hub of lysosomes, it is therefore not surprising that their impairment at different levels causes dysregulation of the lysosome-linked pathways, first endocytosis and lipid metabolism and, above all, autophagy. In fact, lysosomal storage materials have a negative impact on the flux through autophagy and an increasing number of LSDs are described to have autophagic impairment [51]. This is mainly characterized by defects in the vesicular trafficking, inhibition of autophagosome-lysosome fusion and autophagosome formation, reduction in autophagosome and autophagic degradation, defects in autophagosome maturation, increase in the number of autophagosomes, accumulation of autophagic cargoes and reduced organelles turnover [51]. Moreover, in LSDs also lipids, such as cholesterol and sphingolipids, accumulate, finally, causing lysosomal membrane impairment and a failure or improper fusion of lysosomes with autophagosomes [52].

Being lysosomal genes’ housekeeping, it is not surprising that LSDs are generally multisystemic and highly devastating diseases.

Most LSDs have no cure and, where available, the most common treatment, ERT, is not able to help some clinical aspects, as the neurological involvement. Therefore, to identify new therapeutic targets as well as innovative therapeutic strategies it is extremely important to understand the alteration of all the cellular pathways that may be related to lysosomal dysfunction.

To this aim, models like *C. elegans*, *D. melanogaster* and *D. rerio* have been gaining ground in recent years, as they allow to analyse many pathways in *in vivo* contexts, using simpler methods than those used for rodents or other mammals, taking advantage of easier genetic manipulation methods, higher number of animals obtainable and analysable in shorter times, availability of different tools of analysis and, finally, fewer ethical concerns.

## 3. Modelling LSDs in *Drosophila*

### 3.1. Approaches to Model Diseases in Drosophila

*Drosophila melanogaster* is a widely used and highly manageable genetic model organism, useful to understand many molecular and developmental processes involved in human diseases. Many biological, physiological and neurological properties are, in fact, conserved between mammals and the fruit fly. Approximately 75% of human disease-causing genes have a functional homolog in the fruit fly, performing the same function in *Drosophila* tissues as in humans [53,54,55].

**Figure 5:**
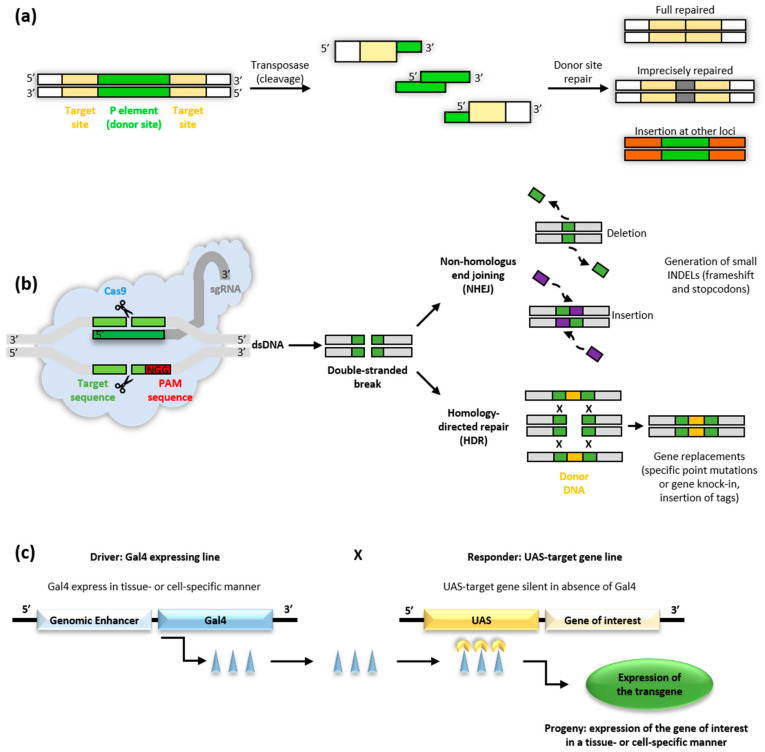
Approaches to model diseases in *Drosophila*. Schematic view of the principal genetic approaches adopted to manipulate the *Drosophila* genome in a reverse approach. (**a**) P-element excision can generate a deletion or a mutation, can exit leaving the region fully intact or produce insertion in other sites. (**b**) The combination of the bacterial endonuclease Cas9 with a small guide RNA (gRNA) can drive the Cas9 to a genomic target site 5′ to an NGG protospacer adjacent motif (PAM). Repair of CRISPR/Cas9-mediated DNA double-strand breaks can occur by error-prone non-homologous end joining (NHEJ) or by homology-directed repair (HDR). (**c**) The UAS-GAL4 binary system allows the expression of the desired construct under the control of GAL4 protein. The system combined two transgenic lines, the driver line carrying the GAL4 expressed under a genomic enhancer and the responder line carrying the UAS promoter upstream of a gene of interest.

*Drosophila* offers a good background for genetic and biological studies of different pathological conditions such as neurological, cardiac and metabolic disorders [53]. To understand the function of the gene of interest generally the inverse genetics approach is used, creating mutations in fly homologs of human genes to study their phenotypes *in vivo*. Several strategies are available to knockout genes in *Drosophila*: transposon-mediated mutagenesis and excision of existing transposable elements (TE), FLP/FRT (recombinase flippase) recombination system, phiC31 integrase-mediated targeted insertion and, more recently, targeted gene disruption using clustered regularly interspaced short palindromic repeats/Cas9 (CRISPR/Cas9) mutagenesis. In parallel, gene silencing and knockdown are possible by the RNA interference (RNAi) approach, taking advantage of the GAL4/UAS system. In addition, to loss-of-function studies, wild-type or mutant version of a human disease-causing gene (transgene) can be overexpressed in flies to evaluate the gain of function effects, also in specific tissues [53,56] (Figure 5).

#### 3.1.1. Transposable Elements

P-elements are the most used transposable elements in *Drosophila* for exploring genes function (Figure 5a). P-element insertions are used as starting-points for generating chromosomal deletions to remove flanking genes, by screening for imprecise excision events or by selecting for male recombination events. They are classified in autonomous or non-autonomous. Autonomous P-elements encode a functional own transposase essential for their mobilization by a cut-and-paste mechanism inside a genome; non-autonomous ones need an external source of transposase to be mobilized. During these years, many transposon vectors have been developed such as piggyback and Minos, and diverse systems, like FLP/FRT recombination and phiC31 integrase-mediated targeted insertion, have been used to target specific genes in the fruit fly genome. Moreover, many engineered vectors have been developed for diverse transgenic applications like gene disruption, enhancer trapping, gene-tagging, targeted misexpression, RNA interference and inducible gene expression or repression [57,58].

#### 3.1.2. The CRISPR/Cas9 Approach

The CRISPR/Cas9 is an innovative and powerful system to manipulate the genome of various organisms in an easy and precise way. For more than 30 years, CRISPR systems has been known to provide adaptive immunity to bacteria and archea, and only in the last 10 years it has been applied to genome manipulation, also in *Drosophila* [59]. This approach consists of the bacterial endonuclease Cas9 and a small guide RNA (gRNA, drawable on the gene of interest) which drives Cas9 to a genomic target site 5′ to an NGG protospacer adjacent motif (PAM). Repair of CRISPR/Cas9-mediated DNA double-strand breaks can occur by error-prone non-homologous end joining (NHEJ) or by homology-directed repair (HDR) (Figure 5b). Using the NHEJ approach it is possible to obtain insertions/deletions in selected regions of the gene of interest. Using the HDR, the homologous recombination allows repairing the double-strand break with the desired piece of exogenous DNA, for example a mutated form of the gene of interest. This new genetic tool allows the generation of complete loss-of-function or null mutations in all genes of *Drosophila* genome, making specific mutations within a coding sequence to model the effects of precise genetic mutations (knock-in), exploring the function of a specific protein domain, characterizing splice sites and fusing specific reporters (GFP, YFP) at exact locations within a gene [60,61,62]. Being relatively simple to draw and generally requiring fewer generations of flies than systems with transposons to obtain the desired mutant, CRISPR/Cas9 is increasingly used, especially in the creation of new disease models in the fruit fly.

#### 3.1.3. The GAL4/UAS System

One of the most important genetic systems used in the fruit fly is the GAL4/UAS (Figure 5c). This approach allows driving the expression of a gene in a specific/defined way for studying its expression and function [63]. This system consists mainly of two parts: the *GAL4* gene, coding for the GAL4 transcription activating protein in yeast and the UAS (Upstream Activation Sequence), a short sequence of the promoter to which GAL4 binds to activate the transcription [63].

Many fly stocks carrying the UAS in the promoter region of the gene of interest are available or can be easily generated. The GAL4 gene has been inserted in many positions in the *Drosophila* genome, generating a lot of “enhancer-trap” line specific for different cells and tissues. Therefore, the expression of a gene of interest can be easily driven, upregulated or downregulated simply crossing an enhancer-GAL4 line with the fruit fly line expressing the sequence of interest downstream of the UAS. [56,61,64,65].

The GAL4/UAS is a powerful genetic tool and it can be used for many approaches in *Drosophila*. In the last decades, researchers have taken advantage of this system. They have inserted P-elements containing cellular markers, as GFP or Beta-galactosidase (LacZ), after an endogenous promoter; or introduced transposable elements to generate cell-type-specific GAL4 expression lines. One of the main uses of the GAL4/UAS system is the RNA interference (RNAi) approach, which consists in expressing short repeat RNA hairpins targeting specific genes, to knockdown their mRNA levels. In *Drosophila*, RNAi is a powerful tool to study protein-coding genes for phenotypes of interest. Indeed, researchers can use the GAL4 driver lines and the UAS-RNAi responder lines to investigate the function of genes in cellular and developmental processes from embryos to adults [61].

### 3.2. Tools to Study LSDs’ Pathways in Drosophila

Thanks to all these transgenesis methods, *Drosophila melanogaster* has become a powerful and versatile animal model, easily used to create disease models and to study cellular pathways, as the endolysosomal and the autophagic ones. In the last few years, many biochemical, molecular, genetic and imaging approaches have been developed to study these pathways.

#### 3.2.1. Reporter Lines

One of the most used tools in *Drosophila* is the GAL4/UAS system. Thanks to the fruit fly scientific community, over the years many reporter lines have been developed and made available through public stock centres (i.e., Bloomington *Drosophila* Stock Center, Vienna *Drosophila* Resource Center, Kyoto Stock Center). UAS-lines expressing GFP and/or mCherry-tagged genes have been developed for a lot of Atg and Rab proteins, as well as for Lamp (lysosomal associated membrane protein). In particular, GFP and/or mCherry-tagged Atg8a are largely used to detect the autophagic structures (including autophagosomes and autolysosomes); reporter lines expressing other tagged-Atg proteins (Atg5 and Atg12) allow the detection of phagophores; GFP and/or mCherry-tagged Lamp lines identify lysosomes and autolysosomes; the combination of GFP-Lamp1 with mCherry-Atg8a allows to distinguish autophagosomes from autolysosomes; tagged-Rab proteins (i.e., Rab5, Rab7, Rab11) allow to follow the endocytic pathway and the maturation of endosomes [29]. All these lines, crossed with proper enhancer-GAL4 lines, allow to analyse the endolysosomal and autophagic pathways very easily in a cell- and tissue-specific manner using the confocal microscopy. For example, crossing this reporter lines with elav-GAL4 or repo-GAL4 enhancer flies, it is possible to analyse these pathways specifically in neurons and glial cells respectively. Among others, this tool could be useful to better understand the alterations of the different CNS cells in diseases with neurological involvement, such as many LSDs. Many UAS-lines have been developed also to overexpress or downregulate (thanks to RNAi approach) all these genes. This is very useful to understand the implications of up- or down-regulation of a gene of interest in the pathogenic mechanisms of a disease.

#### 3.2.2. Vital Dyes and Antibodies

Alongside the GAL4/UAS approach, other systems have been developed that take advantage of confocal microscopy for evaluation of endolysosomal and autophagic pathways. The most used are definitely vital dyes and antibodies. Among these, LysoTracker^®^ and acridine orange are membrane-permeable dyes that accumulate in acidic organelles allowing the detection of lysosomes. Magic Red is a dye marking lysosomes/autolysosomes containing active cathepsin [29]. The LysoSensor™ probes exhibit a pH-dependent increase in fluorescence intensity upon acidification, allowing to investigate the acidification of lysosomes and alterations of their function or tracking. Few antibodies are available to study endogenous proteins in *Drosophila*. However, in recent years toolkits were generated to study autophagy and the endolysosomal pathway, like the one generated in Sean Munro’s lab [66].

#### 3.2.3. Other Tools

Together with reporters, vital dyes and antibodies, other tools allow studying autophagy and endolysosomal pathways in the fruit fly. Electron microscopy allows the identification of autophagic structure and lysosomes on the ultrastructural level [29]. Real-time qPCR permits the analysis of these pathways from a molecular point of view.

Autophagy can be studied in *Drosophila* by treating both larvae and adults with autophagy-modulating drugs, such as rapamycin, ecdysone and spermidine. Feeding larvae with Chloroquine inhibits acidification of lysosomes, whereas Bafilomycin affects both autophagosome-lysosome fusion and acidification.

### 3.3. Next-Generation Analysis and Metabolic Studies

To date, next-generation sequencing, lipidomic and metabolomic approaches have been extensively applied to studies conducted in mouse models and human samples. However, these techniques can be applied also to easier models like *Drosophila* to unravel, by high throughput systems, genes, proteins and metabolites involved in pathways of interest. Thanks to the wide knowledge of *Drosophila* organ-systems and to the functional analogues to vertebrate counterparts, the fruit fly has been used in the last decade to study the metabolism [67]. Signalling pathways like cell growth, proliferation and death and energy homeostasis, like carbohydrate and lipid metabolisms, are highly conserved in the fruit fly. Therefore, *D. melanogaster* has emerged as a good model to study cellular metabolism, also applying high throughput systems such as nuclear magnetic resonance (NMR) spectroscopy and mass spectroscopy (MS) [67]. Different studies have been conducted in the last decade on oxidative stress, gluconeogenesis, amino acid synthesis, metabolic profile throughout the life cycle, as well as neurotransmitters, amino acids, carbohydrates and fatty acids in metabolic and neurodegenerative fruit fly disease models [67]. All these pathways have been found to be involved to some extent also in LSDs and a deeper metabolomic study by using the fruit fly models could be useful to dissect their involvement in the pathogenesis of these disorders.

## 4. Current *Drosophila* Models of LSDs: An Unexplored Potential

Many *Drosophila* models have been generated for Lysosomal Storage Disorders in the last ten years. Some of them have been analysed in detail, others have only been characterized, leaving great potential for use. Here, we will briefly list the LSD fly models so far generated and the related investigations and phenotypes associated to each of them (Table 1).

Almost all models well reflect the corresponding human pathology. Moreover, quite all of them present affected endolysosomal and autophagic pathways at different levels. In some models, imbalances in the lipid pathway were also detected.

Of note, quite all models have been used to study the neurological pathology, showing abnormalities that go from dysfunctional motility, to abnormal axonal trajectory, decreased number of neuromuscular junction (NMJ) boutons, loss of dopaminergic neurons, as well as apoptosis and increased autophagy at neuronal and glial cells level.

### 4.1. Mucolipidosis Type IV (MLIV) Drosophila Model

MLIV is an autosomal recessive lysosomal storage disease caused by loss-of-function mutations in the *MCOLN1* gene, coding for the potential channel protein mucolipin-1 (TRPML1), resulting in abnormal transport of the lipids to the lysosomes where heterogeneous materials (phospholipids and gangliosides) accumulate [68,69]. The disease is characterized by psychomotor delay, ophthalmologic abnormalities (corneal opacities, retinal degeneration and delayed visual development) and reduced language functions [70].

The *Drosophila Trpml* homolog (*CG8743*) shares 40% amino acid identity with the human protein and is widely expressed, though at low levels. *Drosophila* model for MLIV was generated through a P-element insertion, 242 bases 5′ of the translation initiation site [71]. The mutant flies showed reduced viability due to pupal semi-lethality and a progressive loss of motor function. Mutants’ brain showed progressive signs of neurodegeneration through apoptosis (accumulation of large vacuoles in the brain and detection of apoptosis in both neurons and glial cells by TUNEL assay). A progressive loss of photoreceptors in ommatidia resembles the ophthalmologic abnormalities in human pathology. All mutant tissues showed an increased number of lysosomes and increased lysosomal storage of lipofuscin, the latter sign of disrupted autophagy, with a subsequent increase in autophagosomes and amphisomes (from the fusion of autophagosomes and endosomes). Despite the fusion of lysosomes and autophagosomes, a defect in lysosomal degradation due to over-acidification of the lysosomal lumen, as a result of the loss of *trpml*, was noticed. Inhibition of autophagy leads to an accumulation of damaged mitochondria and to oxidative stress that can induce apoptosis and, therefore, neurodegeneration [71,72]. The *trpml1 Drosophila* mutant also showed a decreased number of synaptic boutons at the NMJ, due to the diminished activation of the Rag/mTORC1 signalling pathway, fundamental for the normal development of the NMJ [73]. A protein-supplemented diet and the inhibition of ALK (tyrosin-kinase receptor, which represses the neuronal amino acids uptake) recover the lethal phenotype and the number of synaptic boutons [73]. Onyenkoke and colleagues, suggest that loss of activity of AMPK (5′ AMP-activated protein kinase) leads to the lack of inhibition of the enzyme target of rapamycin (TOR), a nutrient-sensitive protein kinase, which in turn may inactivate through phosphorylation TRPLM1 channel and, therefore, functional autophagy [74].

### 4.2. Batten Disease/Neuronal Ceroid Lipofuscinose (NCL) Drosophila Models

The neuronal ceroid lipofuscinoses (NCLs) are a group of lysosomal storage diseases, also known as Batten disease, most of all inherited in an autosomal recessive manner. They present variable ages of onset (congenital, infantile, late infantile, juvenile, adult and late adult) and the clinical features of childhood forms are progressive visual loss, mental and motor degeneration, seizures and premature death. Autofluorescent, electron-dense, periodic acid-Schiff (PAS)- and Sudan black B-positive granules accumulate in the cytoplasm of the cells, especially in the lysosomes of brain cells, leading to progressive loss of neurons [75]. NCLs are a group of ten different disorders and they are classified according to the designation of the mutated gene [76].

#### 4.2.1. Palmitoyl Protein Thioesterase I (CLN1) *Drosophila* Model

*CLN1* gene encodes for the lysosomal enzyme palmitoyl-protein thioesterase 1 (PPT1) and mutations in this gene cause the Infantile NCL (INCL), the most severe form of NCL. The predicted gene in *Drosophila* has been identified as *CG12108*, encoding a protein 55% identical and 72% similar to human Palmitoyl-protein Thioesterase 1 and ubiquitously expressed. PPT1 enzyme activity was detected in all tissues in *Drosophila*, although at significantly lower levels than those observed in mice or humans [77].

The *Ppt1* deficient fly generated through RNAi technique displays, unlike the unregular granular deposits of the human’s pathology, homogenous deposits, spherical shaped and composed of thousands of concentric layers of material, similar to the multilamellar bodies observed in Niemann–Pick disease [78]. The osmiophilic deposits are especially located near the cell nucleus and increase in number with age. Abnormal deposits are seen also in third-instar larvae brains. Adult flies are vital and fertile but have a reduced lifespan. No alterations in brain structure were observed as well as no signs of neurodegeneration or apoptosis [78]. Embryos do not display any sign of accumulation bodies; however, motoneurons have abnormal axonal trajectory and cells display aberrant fate specification. Axon defects may mean that Ppt1 is necessary for axon guidance and fasciculation [79].

#### 4.2.2. CLN3 *Drosophila* Model

*CLN3* encodes a transmembrane protein whose function is still unknown. Mutations in the *CLN3* gene are responsible for the juvenile form of NCL [80]. The *Drosophila* orthologue (*CG5582*) encodes a protein which shares many of the properties of human CLN3 and is expressed throughout the fly, partially localized to late endosomes or lysosomes [81].

The *cln3* fly mutant was generated by imprecise excision of a Minos transposable element inserted within the large first intron of the gene [82]. The mutant flies are viable and fertile without developmental abnormalities or accumulation of autofluorescent material in the brain, but they are hypersensitive to oxidative stress compared with control flies. When exposed to conditions of oxidative stress, mutant flies show an accumulation of ROS, which may be responsible for neural degeneration [82]. Another RNAi line against *Drosophila cln3* showed a diminished synaptic growth, similarly to *Trpml* loss of function flies [73].

#### 4.2.3. CLN4 *Drosophila* Model

*CLN4* gene encodes the synaptic vesicle protein CSPα (Cystein-String Protein α). Mutations in this gene cause the only autosomal dominant form among the NCLs [83,84]. Imler and colleagues [85] generated two fruit fly models of CLN4 through P-element insertion of the mutated human CSPα (hCSPα) in the first model and of the mutated *Drosophila* CSPα (dCSPα) in the second one. The hCSPα in *Drosophila* is correctly palmitoylated and efficiently traffics to exon terminals, where it co-localizes with endogenous dCSPα. In both models, where the mutant protein is selectively expressed in neurons, they observed a dose-dependent lethality and reduction of lifespan. From a biochemical point of view, they observed the formation of SDS-resistant, high molecular weight hCSPα oligomers in *Drosophila* neurons. Moreover, they observed that the mutant hCSPα is reduced at synaptic boutons and accumulate in axons and, at the same time, it co-accumulates with Lamp1-GFP and HRS-positive endosomes. This suggested an accumulation of the mutated protein on pre-lysosomal endosomes, that are inefficiently processed for lysosomal fusion. The mutant hCSPα is also enriched in ubiquitinated oligomers. Finally, they observed abnormal membrane structures. Expression of the normal hCSPα restores adult lifespan, meaning that the protein has a conserved function in the fly and expression of the normal dCSPα only partially rescues adult lifespan. Therefore, they concluded that both models replicated the key hallmarks of the human pathology, while the study of these models allowed to find new insights into mechanisms underlying the pathology [85].

#### 4.2.4. Cathepsin D (CLN10) *Drosophila* Model

*CLN10* gene encodes the lysosomal aspartic endo-protease Cathepsin D, ubiquitously distributed in lysosomes. Its main function is to degrade proteins and activate precursors of bioactive proteins in pre-lysosomal compartments.

The *Drosophila* homolog of cathepsin D (*cathD*) encodes a predicted protein, which shares 50% amino acid identity and 65% similarity with human cathepsin D. The mutant fly was generated through the insertion of an EP-element in the *cathD* gene and a successive imprecise excision [86]. The mutant flies are fertile and viable with no differences in lifespan compared to controls. They show a progressive accumulation of storage material in brain, fat body and intestine, which resembles the human pathology and progressive neurodegeneration with the loss of neurons through apoptosis [86]. Finally, the mutant flies show a moderate retinal neuronal loss or vacuolization when compared with the control flies [87].

### 4.3. Mucopolysaccharidosis Drosophila Models

Mucopolysaccharidoses (MPSs) are a group of lysosomal storage diseases caused by deficit of lysosomal enzymes degrading glycosaminoglycans (GAGs). Accumulation of GAGs in several cell types and the consequent secondary cascade of events leading to the dysfunction of other cellular pathways, cause progressive multi-organ impairments [88]. Up to date, 11 different MPSs are known, each one due to mutations in genes coding for lysosomal hydrolases, involved in the degradation of different GAGs, at different steps. All together MPSs incidence varies in the different countries/ethnicities from 1.04 to 4.8 per 100,000 live births [89].

#### 4.3.1. Mucopolysaccharidosis Type II (MPS II) *Drosophila* Model

Iduronate 2-sulfatase (IDS) is a lysosomal enzyme involved in the degradation of the glycosaminoglycans heparan- and dermatan-sulfate. Mutations in the *IDS* gene lead to a lack of function of the enzyme and are responsible for Mucopolysaccharidosis type II (MPS II, Hunter Syndrome), an X-linked recessive LSD [90,91]. Main clinical features of MPS II are short stature, skeletal deformities with enlarged head, organomegaly, cardiac and respiratory diseases and, in the severe forms, a progressive neurological involvement [92].

In *Drosophila* a unique homologous gene (*CG12014/Ids*) is present in the third chromosome and the protein shares 47% identity with human IDS [93]. Recently, ubiquitous, pan-neuronal and glial fruit fly knockdown model for MPS II has been developed by an RNA interference approach. However, glycosaminoglycan storage, locomotion behaviour and molecular markers for endolysosomal and autophagic pathways resulted not affected. This suggested that a strong, but not completely abolished IDS-activity is not enough to induce a fly pathological phenotype, suggesting the need for a total knockout *Drosophila* model [94].

#### 4.3.2. Mucopolysaccharidosis Type IIIA (MPS IIIA) *Drosophila* Model

Mucopolysaccharidosis type IIIA (MPS IIIA) is an autosomal recessive LSD due to a mutation in the gene of *N-sulfoglucosamine sulfohydrolase* (*SGSH*) that causes a lack of activity of this enzyme, involved in the degradation of the glycosaminoglycan heparan-sulfate. The accumulation of heparan-sulfate leads to a dysfunction of lysosomes and to the onset of the pathology [95]. The main clinical feature is a severe central nervous system degeneration, together with mild somatic symptoms. Common features are hyperactivity with aggressive behaviour, delayed development, sleep disorders, delayed speech development (or no speech at all) and severe cognitive retardation [96].

The homologous of human *SGSH* has been identified in *D. melanogaster* with the gene *CG14291*, with which it shares 53% identity. The fruit fly model for MPS IIIA has been generated by the knockdown of *CG14291* through RNA interference [97]. This model presents a significant accumulation of heparan-sulfate in 1-day old whole flies, which further increases at 6 weeks of age. The specific neuronal knockdown of *CG14291* leads to a significant defect in climbing activity, worsening with age, a hallmark of nervous system dysfunction and neurodegeneration. This specific knockdown also showed an increased number of acidic vesicles and the disruption of the vesicular trafficking (with impaired autophagic activity) that may contribute to neuronal dysfunction [97].

#### 4.3.3. α-N-acetylglucosaminidase (NAGLU) Homologous Identified in *Drosophila*

α-N-acetylglucosaminidase (NAGLU) is a lysosomal enzyme involved in the degradation of the glycosaminoglycan heparan-sulfate. NAGLU deficiency leads to a progressive accumulation of partially degraded heparan-sulfate and to the onset of Mucopolysaccharidosis type IIIB (MPS IIIB), an autosomal recessive LSD [95]. MPS IIIB is characterized by delayed development and progressive neurodegeneration, although the pathology seems to advance slower and to be less severe than MPS IIIA [96]. Comparative studies of NAGLU showed highly protein sequence conservation among species and also in *Drosophila* (41% identity) [98].

#### 4.3.4. Mucopolysaccharidosis Type VII (MPS VII) *Drosophila* Model

Mucopolysaccharidosis type VII (MPS VII) is an autosomal recessive LSD due to a deficiency in the activity of the lysosomal enzyme β-glucuronidase (β-GUS). The lack of activity of β-GUS leads to the accumulation of partially degraded dermatan-, heparan- and chondroitin-sulfate in the lysosomes of many cells and tissues and to the onset of diverse clinical features in patients, such as short stature, cognitive disability, skeletal abnormality, motor impairment, hernias, hepatosplenomegaly, hydrops fetalis and cardiac and respiratory problems [96].

The *Drosophila* model of MPS VII has been generated by the knockout of the *β-GUS* orthologue (*CG2135*, in the Flybase, also known as *βGlu*) [99]. The fruit fly sequence shares >40% identity and >60% similarity with that of the human β-GUS protein and the active site amino acids of the human β-GUS are conserved in *βGlu* protein [100]. The activity of βGlu is 3.7 × 10^6^ units/mg, comparable to the activity of human β-GUS [101,102].

The adult knockout fly exhibited typical features of MPS VII, with reduced lifespan (5 to 13 days) and progressive decline in locomotor activity (85% decline in climbing activity at the fourth week of age). In the brain of adult flies, an increased number and size of lysosomes was observed. Furthermore, the *CG2135*-/- model showed an abnormal increase in the number of ubiquitinated proteins and in the number of mitochondria, which might be caused by a defect in the lysosome-mediated cellular pathway. In brain sections, there is an appearance of cellular vacuolization and loss of dopaminergic neurons, which are involved in locomotor activity. Finally, the loss of muscular fibers integrity of thoracic muscles through apoptosis of myocytes could be another pathological basis for locomotor disability. Of note, neuromuscular degeneration and locomotor deficit are attenuated by treatment with resveratrol [99].

### 4.4. Sphingolipidoses Drosophila Models

Sphingolipidoses are a group of inherited lysosomal storage diseases characterized by a massive sphingolipid and membrane accumulation in lysosomes, neurodegeneration and short life expectancy [103].

Sphingolipidoses include GM1 and GM2 gangliosidosis, Niemann–Pick disease, Globoid cell leukodystrophy (Krabbe disease), Gaucher disease, metachromatic leukodystrophy and Fabry disease.

#### 4.4.1. Niemann–Pick Type C Disease (NPC) *Drosophila* Model

NPC is an autosomal recessive lysosomal storage disorder caused by mutations in the genes *NPC1* and *NPC2* [104,105] that determine a defective cholesterol transport from the lysosomes. Free cholesterol, sphingolipids and gangliosides accumulate in lysosomes of all cells leading to hepatosplenomegaly and neurodegeneration [106,107]. The co-accumulation of cholesterol and glycolipids forms detergent-resistant membranes whose cluster leads to conversion into multilamellar bodies [108].

Two homologous *NPC1* genes were identified in *Drosophila melanogaster*: *dnpc1a*, which shares 44% similarity and 63% identity with human *NPC1* and *dnpc1b*, which shares 55% similarity and 38% identity with human *NPC1* [109].

Meanwhile, the *NPC2* homologous gene in *Drosophila melanogaster* is represented by a family of eight genes named *npc2a-h*, where *npc2a* is the one with the highest sequence identity (36%) and similarity (53%) to human *NPC2* [110].

The first *Drosophila* model for NPC was generated through RNAi for *dnpc1a* [109]. This gene is ubiquitously expressed in embryo and third-instar larva, but when mutated causes death at first larval instar stage. First-instar larvae exhibit sterol accumulation like that of mammalian NPC cells. Food supplementation with 7-dehydrocholesterol and ecdysone, which is the hormone needed for the eclosion, or dnpc1a specific expression in ring gland, the site of ecdysone synthesis, rescue the lethal phenotype and the mutants can reach the adult stage. The adult flies show sterol accumulation in brain and presence of multi-lamellar organelles in Malpighian tubules cells, which resembles the human pathology [109,111].

The cholesterol-rescued adult mutants have strong locomotor defects worsening with age and a reduced lifespan. In adult heads, a threefold increase of free cholesterol compared with wild-type adults is present, forming aggregates in neurons already at 5 days of age. Adult brains and retina present a progressive vacuolization, that is sign of neurodegeneration, although without apoptosis. Neurons are enriched with multi-lamellar bodies that progressively accumulate with age. Finally, adult mutants present a much more elevated age-dependent loss of phototransduction [112].

The second *Drosophila* model for NPC was generated through P-element imprecise excision of the gene *npc2a*, which is the most ubiquitously expressed in the family of *npc2* gene [110]. The mutation is not lethal, and flies reach adult stage and have good fertility, although with a reduced lifespan. Sterols accumulate in all tissues and multi-lamellar and multi-vesicular bodies are increased in Malpighian tubules. In adult brains, there is absence of vacuolization, but there are many TUNEL-positive cells.

The third *Drosophila* model for NPC was generated through P-element imprecise excision of the gene *npc2b*, which is expressed specifically in tracheal system and hypopharynx. This mutant also reaches the adult stage and is fertile but shows no accumulation of sterol in any tissue. The *npc2a/npc2a; npc2b/npc2b* double mutant is, on the other hand, lethal and animals die at larval or pupal instar, with only one-tenth of flies reaching the adult stage. The brain presents an elevated number of TUNEL-positive cells, most of them neurons [110].

#### 4.4.2. Gaucher Disease (GD) *Drosophila* Model

Gaucher disease (GD) is a rare autosomal recessive disease belonging to lysosomal storage disorders. It is characterized by mutations in the *β-glucocerebrosidase* (*GBA*) gene that encodes a lysosomal enzyme catabolizing glucocerebroside, which accumulates into lysosomes of several cells and tissues [113].

Clinical manifestations of GD are visceral and skeletal abnormalities, with hepato-splenomegaly, pathological macrophages infiltration in the lung, osteonecrosis that causes osteoporosis and, in the severe forms (Gaucher disease type II and type III), neurological dysfunctions [114].

*Drosophila* genome has two *GBA1* homologous genes, *CG31148* (*dGBA1a*) and *CG31414* (*dGBA1b*) that show differential tissue expression: dGBA1b protein is expressed in the adult brain and in the adult fat body, whereas dGBA1a is mainly expressed in the adult fly digestive system and not in the adult brain [115].

Three different *Drosophila* mutants for GBA1 were generated using homologous recombination: *dGBA1a* mutant, *dGBA1b* mutant and *dGBA1a,b* double mutant: only *dGBA1a* flies showed a significantly increased survival compared with control flies, whereas *dGBA1b* mutant and *dGBA1a,b* double mutant showed a significantly reduced lifespan compared with control flies, both in normal and in low nutrient condition, together with developmental defects from larva to pupa and from pupa to adult [116,117,118]. *dGBA1b* mutant and *dGBA1a,b* double mutant showed progressive age-dependent locomotor deficits in climbing ability, starting from 5 days after eclosion [116,118] and reduced fertility, with age-dependent declined number of laid eggs per female. As in human pathology, *dGBA1b* mutant and *dGBA1a,b* double mutant showed lack of enzyme activity and significant accumulation of glycosylceramide in their heads [116,119], with subsequent increase in lysosomes number and size, which leads to a dysregulation of autophagy and the further accumulation of undegraded poly-ubiquitinated protein in whole flies and in heads [117,119,120,121]. The dysregulation of autophagy, whose accumulation of Ref(2)p is a marker [117], also leads to the accumulation of giant and dysfunctional mitochondria and a subsequent hypersensitivity to oxidative stress. Elevation in levels of *Hsc70-3* (Heat shock 70-kDa protein cognate 3, orthologue for mammalian BiP, endoplasmic reticulum chaperone BiP) mRNA, Xbp1 (X-box binding protein 1) splicing and in the level of phosphorylated eIF2α (eukaryotic translation initiation factor 2α) in mutated flies are markers of activation of Unfolded Protein Response pathway and of stress of endoplasmic reticulum [118,119,122]. Moreover, loss of GBA1 in brain leads to neuroinflammation and neurodegeneration (increased brain vacuolization) and loss of synaptic functions [117,119,120,121]. Treatment with the pharmacological chaperone ambroxol decreases the levels of UPR parameters, ameliorates the inflammation and the neuroinflammation and increases the lifespan, although it neither rescues the enzyme activity nor reduces substrate accumulation [119].

In the last few years, GD *Drosophila* model has been used to demonstrate the association between mutated *GBA* gene and increased risk to develop Parkinson’s disease. In particular, *Drosophila* double heterozygous for mutated *GBA* gene, as well as *Drosophila* with the insertion of the mutated human *GBA* gene, shows activation of the UPR pathway (like human carriers of mutated gene) and parkinsonian signs, such as loss of dopaminergic neurons and aggregation of α-synuclein. These models also show progressive defective locomotion and a shorter lifespan [123,124].

#### 4.4.3. Metachromatic Leukodystrophy (MLD) *Drosophila* Model

Metachromatic leukodystrophy (MLD) is an autosomal recessive LSD caused by mutations in the gene ARSA, encoding for the lysosomal enzyme Arylsulfatase A, which catalyses the conversion of sulfatide (sulfogalactosylceramide) in galactosylceramide. Lack of this enzyme activity leads to the accumulation of sulfatides into lysosomes in central and peripheral nervous system, leading to a progressive demyelination and reduced nerve conduction velocity [125]. MLD comprises three clinical subtypes, depending on the age of symptoms onset: late-infantile (before 30 months of age), juvenile (between 2.5 and 16 years of age) and adult (after 16 years of age) forms. It is characterized by deterioration of motor and cognitive functions or behavioral problems [126]. Lee and colleagues have demonstrated that ARSA, despite being a lysosomal protein, directly interacts with α-synuclein present in the cytosol of the cell, acting like a molecular chaperone: some ARSA disease-causing variants are responsible for a reduction of this interaction and a consequent α-synuclein aggregation, whereas other variants, known to be protective against parkinsonism, encode for proteins that have a stronger interaction with cytoplasmatic α-synuclein [127]. In this last publication [127], the authors generated a transgenic fly model for mutant α-synuclein that displays progressive locomotor deficit, reversed by the expression of wild type human ARSA protein, as well as of the human ARSA N352S variant (protective), but not by the expression of the L300S variant (pathogenic).

#### 4.4.4. Fabry Disease *Drosophila* Model

Fabry disease is a recessive X-linked LSD due to mutations in the gene coding for the lysosomal enzyme α-galactosidase A (α-Gal A), this leads to the progressive accumulation of globotriaosylceramide (GB3) and its acylated form lyso-GL3 (lyso-GB3) in endothelium [128]. Clinical manifestations include progressive renal insufficiency, cardiac impairment, gastrointestinal system pathology and neuropathology mainly referred to cerebral vasculopathy [129]. Fabry disease is classified, based on the age at onset, in classic, which presents onset between the first and the second decade of life and late onset, which is characterized by varied and later onset and symptomatology [130]. Patients often present vascular remodeling with an increased thickness of the carotid intima-media, due to the huge proliferation of smooth muscle cells [131,132]. Recently, a new *Drosophila* model for Fabry disease was generated by integrating wild type and mutant *α-Gal A* gene under the control of UAS/GAL4 system in the fly genome [133]. Adult flies expressing mutated α-Gal A display significant locomotor dysfunction and shortened lifespan, compared to flies expressing the wild type form. Biochemically, they saw that mutated variants were retained in ER and underwent Endoplasmic-reticulum-associated protein degradation (ERAD), with the consequent activation of the UPR machinery (increase of *Hsc70-3*, spliced *Xbp1* and *Atf4* (Activating Transcription Factor 4) mRNA levels). Moreover, selective expression of mutated variants in dopaminergic cells, lead to cell death. Treatment of flies from first day of eclosion for 22 days with migalastat, a molecular chaperone, rescues lifespan, locomotor defects and dopaminergic cells deaths, but fails in ameliorating UPR parameters [133].

#### 4.4.5. Saposin Deficient Sphingolipidoses *Drosophila* Model

Saposins are lysosomal soluble hydrolases derived by enzymatic cleavage from the precursor prosaposin. Mutations in *prosaposin* gene cause ubiquitous storage of sphingolipids in humans [134].

The *Drosophila* orthologue of *prosaposin* is called *Saposin-related* (*Sap-r*) and encodes a protein very similar to the human prosaposin. Sap-r in *Drosophila* is ubiquitously expressed, especially in metabolic organs, central nervous system (in both neurons and glia) and embryonic hemocytes. It is localized in endosomes, lysosomes and autophagosomes, like mammalian prosaposin. Two models were developed: the first one through FLP-FRT based deletion, removing the first three exons of the gene [103], the second one via an imprecise P-element mobilization strategy with deletion of the first two exons [135].

The *Sap-r* model developed by Sellin and colleague [103] presented a semi-lethal phenotype, in fact, only 70% of larvae reach the pupal stage and 55% the adulthood and adult flies have a reduced lifespan. Accumulation of acidic vesicles are seen in almost all organs and they increase in size and number with age. Lysosomal dysfunction leads to a block in the autophagic pathway with a subsequent accumulation of defective mitochondria and elevated H_2_O_2_ levels, sign of increased oxidative stress. In all brain regions are present enlarged multivesicular and multilamellar bodies and a large amount of dead and vacuolized cells, increasing with age, all signs of neurodegeneration. Mutant flies also show a defect in climbing ability that worsen with age and correlates with neurodegeneration, indicating a progressive motor function decline [103,135].

The second *Sap-r* model [135] confirmed perturbations in sphingolipid catabolism leading to reduced longevity and neurodegeneration. The authors also highlighted a swelling of neuronal soma and suggested a possible calcium homeostasis deficit.

### 4.5. LSDs-Like Drosophila Models

LSDs-like are diseases that present tracts similar to LSDs but are not caused by mutations in lysosomal proteins. In this group, disorders of lysosome-related organelles are included [136].

#### Spinster/Benchwarmer *Drosophila* Model

*Spinster* (*Spin*) or *benchwarmer* (*bnch*) gene encodes a transmembrane protein localized to the late endolysosomal compartments, mainly expressed in motoneurons [137,138]. *Spin* does not have any human homologous, therefore, no human pathology is associated to this model. When mutated, *Spin* causes a LSD-like neurodegeneration [137]. The *Drosophila* model for *spinster* shows reduced viability and lifespan with lethality at the late pupal stage [13,138]. Adult escapers exhibit progressive locomotor defects, consisting in difficulty in righting after a fall and lower level of locomotor activity, that worsen with age and result in death within 5–12 days [139].

In *Spin* mutant, autofluorescent material that overlaps with *Spin* gene expression can be identified: neurons and glial cells contain multilamellar bodies and electron-dense lobulated granules very similar to lipofuscin and also the retina contains a large number of abnormal membranous inclusions in the cell bodies of mutant photoreceptor [13,139]. Moreover, an accumulation of enlarged lysosomes with partially degraded contents in *spin* mutant muscle and within the presynaptic nerve terminal was observed [138,139]. The material that accumulates in *Spin* mutant corresponds to ceramides, increased more than 800% on day 1 in mutant brains [137].

In *Spin* mutant a synaptic overgrowth with an increase of more than 200% in bouton numbers and an expansion of total synaptic area was observed, albeit a reduction in muscle fibers dimension was also observed and a deficit in presynaptic transmitter release [138]; in fact, at high stimulation frequency, the amplitude of the excitatory junctional potentials declines to 55–60% of the original response after 10 min [139].

The abdominal ganglion of *Spin* mutant is longer [13] and vacuolated, sign of neurodegeneration; moreover, there is a severe neuronal loss with a significant decrease in the number of neuronal cell bodies and severe vacuolization in cortical brain layers [139]. The extent of these defects is progressive; in fact, there is an increased number and size of retinal vacuoles in an age-dependent manner [139].

## 5. *Drosophila* as a Tool for Drug Testing and Screening

All the above results have led *Drosophila* to be a model mainly used to study basic mechanisms and conserved pathways. However, it remains an underestimated model to address drug efficacy and toxicity and a viable drug screening model. Screening in a living animal as the fruit fly can speed up and reduce the costs of the drug screening process, since it allows an *in vivo* research, much more reliable than the *in vitro* one and not practicable in mice.

The use of fruit fly-based models for drug screening has already led to several positive results on different neurological and cancer models [140]. Moreover, in recent years, several efforts have been made to refine and speed up dedicated techniques, for example by developing *in vivo* large-scale chemical screening platforms, to test up to 2000 compounds using 96-well plates [141].

Till now, *Drosophila* models for LSDs have been used to test single drugs, like resveratrol for MPS VII [99] or ambroxol for Gaucher disease [119]. However, since all LSDs lack therapy for the CNS involvement (and in most cases also for bone and cardiac pathologies), it would be of great importance to exploit *Drosophila* models to carry out drug screening targeted to the CNS and able to cross the blood-brain barrier (BBB) [142,143].

Finally, a further advantage of screening in *Drosophila* of molecules already approved for other therapeutic uses in humans is represented by the possibility, in case of identification of an effective molecule, to actively contribute to drug repurposing, thus avoiding a possible off-label use of the drug and drastically shortening the timing of a correct drug translation to patients.

### 5.1. Drug Delivery in Drosophila

Drugs or small molecules can be delivered in *Drosophila* at all developmental stages, in acute or chronic exposure experiments and for low or high-throughput screens. Drugs can be administered by permeabilization of embryos [144], by adding to the solid media for chronic exposures or in a dilute solution of yeast paste for shorter exposures, as well as by injection when larvae are studied [144,145,146,147,148]. Administration of drugs to adult flies can be delivered in the food, from a sucrose/drug-saturated filter paper or by drug injection into the abdomen, permitting a quick diffusion throughout the organism [149] and also as a vapor (e.g., ethanol, cocaine and anesthetics) [150,151,152,153].

Drug administration through feeding may encounter some problems, such as animal rejection of food containing drugs. In fact, *Drosophila* can respond to a broad range of taste chemicals avoiding the ingestion of toxins or unpleasant compounds. In addition, drugs can affect the molecular targets that modulate feeding behavior [154,155,156]. To determine whether the presence of a drug influences food intake, different feeding assays can be performed, such as the capillary feeder (CAFE), colorimetric dye observations of proboscis extension (PE) and food labelling with a radioactive tracer [157,158,159].

Furthermore, several pharmacological phenotypic screening assays have been developed to expose adult, larva or embryo to liquid, solid or volatile drugs and measure the desired phenotypic change. Usually, in high-throughput screenings, larvae, adults or embryos are dispensed into plates and fed with liquid or solid food, consisting of sugar and yeast extract and supplemented with fluorescein and/or drugs for visualization of food and drug intake. The plates, under well-controlled environmental conditions, can be screened by using imaging techniques (as for cell culture), biochemical approaches (plate reader) or behavioral tests, using camera that records movements [141,156,160,161,162]. The other alternative option is to expose flies to volatile drugs and test the behavioral effect after chemical exposure [163]. Thus, chemical libraries may be tested for their ability to modify or cause a specific phenotype, such as growth defects, lethality, uncoordinated or reduced locomotion and morphological defects in a whole organism [164].

In *Drosophila*, as suggested by Pandey, the physiologically effective concentrations of a drug in the feeding substrate are in the range of 0.01–100 mM [55]. Furthermore, when new compounds are tested in *Drosophila* it is recommended to start with a pilot study using at least three different concentrations at log dilutions in the feeding substrate (for example 0.01, 0.1 and 1.0 mM) and to analyze the efficacy in a particular assay. Following this step, the appropriate concentration, based on the obtained results, can be used for the full screen [55]. After ingestion of food containing drugs and oral adsorption, it is difficult to predict the bioavailability at the site of physiological activity. To examine the actual *in vivo* bioavailability of the drug high-performance liquid chromatography or mass spectrometry methods can be applied to analyze *Drosophila* tissues and obtain highly sensitive chemical quantification after administration [160]. In addition, to drug concentration, it is possible to identify the more efficient exposure timing: exposing multiple samples to the drug at the same time simplifies the exposure-response assessment, speeding up the identification of the correct dosage of the compounds and therefore carrying out the pharmacological screen [55].

Recently, an interesting approach focused on the simultaneous targeting of specific effectors (combination of more drugs at the same time) in cancer and age-related pathologies in *Drosophila*, has suggested a potential application of this model in drug repurposing and poly-therapy development [165,166]. Given that LSDs are biologically complicated disorders, with different intracellular pathways deranged, the effectiveness of a poly-pharmacological approach targeting the different alterations could be investigated in depth.

Taking into consideration the manageability of the fruit fly model, the drug delivery methods available and the potential of the model in drug repurposing and discovery, a wide range of different approaches can be adopted to conduct pharmacological tests on LSDs *Drosophila* models already generated, thus helping the identification of lead compounds likely to be therapeutically useful both in mono- and in combinational therapies.

### 5.2. Drosophila BBB as a Tool for CNS Treatments Discovery

The BBB tightly regulates the movement of ions, molecules and cells between the blood and the brain, allowing the maintenance of the brain homeostasis in response to extrinsic factors and physiological changes [167]. As mentioned above, the drug exclusion properties of BBB are one of the limiting steps to the development of effective CNS disease treatments and model systems able to recapitulate the functions of the BBB are highly attractive. In vertebrates, the brain vascular endothelial cells form the BBB and together with pericytes, myocytes, astrocytes, microglia, extracellular matrix and neurons compose the neurovascular unit (NVU) [168]. NVU functions as a detector for neuronal needs and contributes to the BBB development and integrity [169]. *Drosophila*, by contrast, has an open circulatory system, where hemolymph flows and transports molecules to tissues and organs [170]. As in vertebrate, the *Drosophila* CNS is protected by a BBB, that insulates the CNS from the hemolymph and it is composed by two glial subtypes, the apical perineurial glia (PG) and basal subperineurial glia (SPG) that form collectively the surface glia. Furthermore, apically to the PG, a neural lamella resembles the extracellular matrix surrounding the vertebrate NVU [171]. Despite that, at first glance, the *Drosophila* BBB results anatomically different from that of vertebrates, fruit fly humoral/CNS barrier conserves the xenobiotic exclusion properties typical of the vertebrate vascular endothelium. The semipermeable properties of vertebrate BBB results from the function of different players that can be found, at least in part, in *Drosophila*. Intercellular protein complexes between endothelial cells such as the tight junction (TJ) regulate the paracellular fluid flux in vertebrate, whereas in *Drosophila* similar function is exerted by the septate junction (SJ), present between SPG cells [172]. SPG cells express the drug efflux transporter Mdr65, the equivalent of vertebrates Mdr1/P-glycoprotein, belonging to the ATP-binding cassette (ABC) [173,174]. Moreover, fly surface glia transcriptome identified a huge number of highly expressed genes as ATP-binding cassette (ABC), solute carrier transporters, cell adhesion molecules, as well as metabolic enzymes, signaling molecules and components of xenobiotic metabolism pathways present in vertebrate, pointing out the functional conservation of BBB between the species [175].

An intriguing example of functional parallelism between *Drosophila* and vertebrates, is the glia cell-neuron lactate shuttle process that allows supply of nutrients to neuronal cells. In vertebrates, the astrocyte uptakes the glutamate released from synapses for lactate production via glycolysis which in turn fuel active neurons [176,177]. In *Drosophila*, the neuronal feeding is achieved mainly by the BBB. SPG cells facilitate the absorption of trehalose, the major energy metabolite in *Drosophila*, to produce glucose that is finally, converted to alanine or lactate taken up by neurons [178,179]. The fly glia–neuron lactate shuttle plays also an additional role in neuroprotection under stress: ROS promotes neuronal lipogenesis and in turn neuronal lipid transport to glia is necessary to protect neuron from lipid toxicity [180,181]. Of note, lactate is also emerging as a neuroprotective agent in mammalian [182,183]. The comparison between fly and vertebrates is less simple when neuroinflammation is discussed. BBB permeability defects, a phenotype often linked to neurodegeneration, have been associated to microglia activation and astrocytes dysfunction [184,185,186]. In *Drosophila*, instead of microglia, much more complex, we find the ensheathing glia that expresses key components of the glial phagocytic machinery [187,188].

*Drosophila* is already a widely *in vivo* model used to disentangle the mechanisms and properties as well as dysfunctions of BBB of CNS disorders [143,189,190,191]. Moreover, different approaches are available to evaluate the capacity of drugs to cross the fly BBB, making this model suitable for preclinical drug screening [148,160,192].

## 6. Conclusions

*Drosophila melanogaster* has the longest history as a genetic model system and it is one of the most studied organisms in biological research.

In the last 15 years, several models for lysosomal storage disorders have been developed, mainly to better understand which pathways are compromised as a result of storages, at what level the endolysosomal and autophagic pathways are dysregulated and, above all, what leads to the neurological impairment. Thanks to these models, some steps forward have been made in the understanding of these pathologies, however, most of them have been poorly used and analyzed, still leaving a huge potential for development.

As seen in some LSD models, thanks to the many tools available it is possible to highlight in vivo defects in the fusion of endosomes and lysosomes, increased apoptosis and autophagy, as well as vacuolization and loss of dopaminergic neurons, decreased number of synaptic boutons, neurodegeneration, apoptosis and increased autophagy in neuronal and glial cells.

Glial cells and neurons are an open paradigm in LSDs. In the fruit fly, a selective expression of genes in these cells could clarify basic mechanisms implicated in the neuropathology. Moreover, it would be of great importance to exploit these models also to better understand the involvement in the CNS disease of secondary storages and of poorly studied pathways, as lipid and glycogen metabolism and of ARL.

Preclinical models have the important function to drive the development or not of specific drugs. However, to reduce translational failures we need predictive and robust preclinical models. In this context, the benefit of small animal models has emerged in the last years since several drugs screening in *D. melanogaster*, *C. elegans* and *D. rerio* have been conducted. The improvement of tools and design of experimental research to identify new compounds in these models could help, change and accelerate the passage from preclinical to clinical studies. In this context, *D. melanogaster* could be an extremely valuable tool, due to the degree of conserved biology and physiology between flies and humans. Nevertheless, it needs to be taken into account that pharmacokinetics and pharmacodynamics of small molecules might display many differences between *Drosophila* and humans and this could lead to significant discrepancies in drug plasmatic levels and tissue distribution profiles. As seen in MPS VII and Gaucher fruit flies, LSD *Drosophila* models can be used to test drugs, differential diets and to search for new molecules potentially able to improve the disease phenotype. Compounds can be easily administered to fruit fly embryos, larvae or adults, also overcoming a possible lethality problem, easily exploiting it as a useful phenotypic feature to test the efficacy of drugs in high-throughput screenings.

In conclusion, we suggest a wider use of the *Drosophila* models already developed by the scientific community working on LSDs to better understand these pathologies, as well as to develop other *Drosophila* models, especially for LSDs still lacking a preclinical model, and above all a therapy, thus exploiting the possibility to carry out drug screening and to identify compounds capable of totally or partially resolve the pathological phenotype.

## Figures and Tables

**Table 1:** Lysosomal storage disorder *Drosophila* models.

Disease	Human Gene	Protein	Protein Localization	*Drosophila* Gene	Protein Alignment Data	Model Generation Method	References
Neuronal Ceroid-Lipofuscinosis (CLN) or Batten Disease							
CLN1	*CLN1/PPT1*	Palmitoyl-protein thioesterase 1 (PPT1)	Cytosol; Golgi apparatus; Lysosomal lumen; Nucleus	*CG12108/Ppt1*	72% similarity, 55% identity	RNAi	[78,79]
CLN3	*CLN3*	Transmembrane protein	Endoplasmic reticulum; Early endosome; Late endosome; Golgi apparatus; Golgi membrane; Lysosomal membrane; Mitochondria; Nucleus; Plasma membrane	*CG5582/Cln3*	Data not available	Minos transposable element imprecise excision; RNAi	[73,82]
CLN4	*CLN4/DNAJC5*	Soluble cysteine string protein α (CSPα)	Cytosol	*CG6395/Csp*	Data not available	P-element insertion	[85]
CLN10	*CLN10/CTSD*	Cathepsin D (CTSD)	Lysosomal lumen	*CG1548/cathD*	65% similarity, 50% identity	P-element imprecision excision	[86,87]
Mucolipidosis (ML) and Mucopolysaccharidoses (MPSs)							
MLIV	*MCOLN1*	Mucolipin-1 (TRPML1)	Lysosome membrane; Late endosome membrane; Cell membrane; Phagosome membrane	*CG8743/Trpml*	40% identity	P-element insertion	[71,72,73]
MPS II, Hunter Syndrome	*IDS*	Iduronate 2-sulfatase (IDS)	Lysosomal lumen	*CG12014/Ids*	47% identity	RNAi	[93,94]
MPS IIIA, San Filippo Syndrome type A	*SGSH*	N-sulfoglucosamine sulfohydrolase (SGSH)	Lysosomal lumen	*CG14291/Sgsh*	53% identity	RNAi	[97]
MPS IIIB, San Filippo Syndrome type B	*NAGLU*	α-N-acetylglucosaminidase (NAGLU)	Lysosomal lumen	*CG13397*	41% identity	none	[98]
MPS VII, Sly Syndrome	*GUSB*	Glucuronidase beta (GUSB)	Lysosomal lumen	*CG2135/βGlu*	40% identity, 60% similarity	Homologous recombination	[99]
Sphingolipidosis							
Gaucher disease (GD) or glucocerebrosidase deficiency	*GBA*	Glucosylceramidase beta (GBA)	Lysosomal lumen; Lysosomal membrane	*CG31148/GBA1a* *CG31414/GBA1b*	31% identity, 49% similarity	Minos transposable element insertion; Homologous recombination; Transposon insertion and precise excision; RNAi	[116,118]; [121,122]; [117]; [120]
Niemann Pick disease type 1C (NPC1)	*NPC1*	NPC intracellular cholesterol transporter 1 (NPC1)	Endoplasmic reticulum; Late endosome membrane; Golgi apparatus; Lysosomal membrane; Nuclear envelope; Plasma membrane	*CG5722/Npc1a* *CG12092/Npc1b*	Npc1a: 44% similarity, 63% identity; Npc1b: 55% similarity, 38% identity	RNAi	[109,111,112]
Niemann Pick disease type 2C (NPC2)	*NPC2*	NPC intracellular cholesterol transporter 2 (NPC2)	Endoplasmic reticulum; Lysosomal lumen	*CG7291, CG3153, CG3934, CG12813, CG31410, CG6164, CG11314, CG11315 (Npc2a-h)*	Npc2a: 53% similarity, 36% identity	P-element insertion and imprecise excision	[110]
Metachromatic leukodystrophy	*ARSA*	Arylsulfatase A	Endoplasmic reticulum; Lysosomal lumen	*CG32191*	Data not-available	PhiC31 integrase system	[127]
Fabry disease	*GLA*	α-Galactosidase	Lysosomal lumen	*CG5731*	Data not available		[133]
Saposin deficiency sphingolipidoses	*PSAP*	Prosaposin (PSAP)	Lysosomal lumen; Lysosomal membrane; Plasma membrane	*CG12010 (Saposin-related)*	Data not available	P-element insertion and imprecise excision; FLP-FRT based deletion	[103,135]
LSD-like							
Spinster/Benchwarmer	-	transmembrane protein and putative late-endosomal/lysosomal efflux permease	Late endolysosomal compartment	*CG8428 (spin* or *bnch)*	Data not available	P-element insertion and imprecise excision P-element insertion	[138,139]; [13,137]

## Data Availability

Not applicable.

## References

[1] Platt F.M., d’Azzo A., Davidson B.L., Neufeld E.F., Tifft C.J. (2018). Lysosomal storage diseases. Nat. Rev. Dis. Prim..

[2] Bellettato C.M., Hubert L., Scarpa M., Wangler M.F. (2018). Inborn errors of metabolism involving complex molecules: Lysosomal and peroxisomal storage diseases. Pediatr. Clin. N. Am..

[3] De Duve C., Pressman B.C., Gianetto R., Wattiaux R., Appelmans F. (1955). Tissue fractionation studies. Biochem. J..

[4] Hers H.G. (1965). Inborn lysosomal diseases. Gastroenterology.

[5] Marques A.R.A., Saftig P. (2019). Lysosomal storage disorders–challenges, concepts and avenues for therapy: Beyond rare diseases. J. Cell Sci..

[6] Kingma S.D.K., Bodamer O.A., Wijburg F.A. (2015). Epidemiology and diagnosis of lysosomal storage disorders; Challenges of screening. Best Pract. Res. Clin. Endocrinol. Metab..

[7] Bulfield G. (1980). Inherited metabolic disease in laboratory animals: A review. J. Inherit. Metab. Dis..

[8] Hindle S., Hebbar S., Sweeney S.T. (2011). Invertebrate models of lysosomal storage disease: What have we learned so far?. Invertebr. Neurosci..

[9] Pastores G.M., Torres P.A., Zeng B.-J. (2013). Animal models for lysosomal storage disorders. Biochem..

[10] Favret J.M., Weinstock N.I., Feltri M.L., Shin D. (2020). Pre-clinical mouse models of neurodegenerative lysosomal storage diseases. Front. Mol. Biosci..

[11] Zhang T., Peterson R.T. (2020). Modeling lysosomal storage diseases in the zebrafish. Front. Mol. Biosci..

[12] Sym M., Basson M., Johnson C. (2000). A model for niemann-pick type C disease in the nematode *Caenorhabditis elegans*. Curr. Biol..

[13] Nakano Y., Fujitani K., Kurihara J., Ragan J., Usui-Aoki K., Shimoda L., Lukacsovich T., Suzuki K., Sezaki M., Sano Y. (2001). Mutations in the novel membrane protein spinster interfere with programmed cell death and cause neural degeneration in drosophila melanogaster. Mol. Cell. Biol..

[14] Julian L.M., Stanford W.L. (2020). Organelle cooperation in stem cell fate: Lysosomes as emerging regulators of cell identity. Front. Cell Dev. Biol..

[15] Ballabio A., Bonifacino J.S. (2020). Lysosomes as dynamic regulators of cell and organismal homeostasis. Nat. Rev. Mol. Cell Biol..

[16] Saftig P., Klumperman J. (2009). Lysosome biogenesis and lysosomal membrane proteins: Trafficking meets function. Nat. Rev. Mol. Cell Biol..

[17] Saftig P., Schröder B., Blanz J. (2010). Lysosomal membrane proteins: Life between acid and neutral conditions: Figure 1. Biochem. Soc. Trans..

[18] Appelqvist H., Wa P. (2018). The lysosome: From waste bag to potential therapeutic target. J. Mol. Cell Biol..

[19] Reggiori F., Klumperman J., Maxfield F.R., Willard J.M., Lu S. (2016). Lysosome biogenesis and autophagy. Lysosomes: Biology, Diseases, and Therapeutics.

[20] Matteoni R., Kreis T.E. (1987). Translocation and clustering of endosomes and lysosomes depends on microtubules. J. Cell Biol..

[21] Pu J., Guardia C.M., Keren-Kaplan T., Bonifacino J.S. (2016). Mechanisms and functions of lysosome positioning. J. Cell Sci..

[22] Ba Q., Raghavan G., Kiselyov K., Yang G. (2018). Whole-cell scale dynamic organization of lysosomes revealed by spatial statistical analysis. Cell Rep..

[23] Perera R.M., Zoncu R. (2016). The lysosome as a regulatory hub. Annu. Rev. Cell Dev. Biol..

[24] Bajaj L., Lotfi P., Pal R., di Ronza A., Sharma J., Sardiello M. (2018). Lysosome biogenesis in health and disease. J. Neurochem..

[25] Lawrence R.E., Zoncu R. (2019). The lysosome as a cellular centre for signalling, metabolism and quality control. Nat. Cell Biol..

[26] Zeigerer A., Gilleron J., Bogorad R.L., Marsico G., Nonaka H., Seifert S., Epstein-Barash H., Kuchimanchi S., Peng C.G., Ruda V.M. (2012). Rab5 is necessary for the biogenesis of the endolysosomal system in vivo. Nature.

[27] Huotari J., Helenius A. (2011). Endosome maturation. EMBO J..

[28] Rink J., Ghigo E., Kalaidzidis Y., Zerial M. (2005). Rab conversion as a mechanism of progression from early to late endosomes. Cell.

[29] Lőrincz P., Mauvezin C., Juhász G. (2017). Exploring autophagy in Drosophila. Cells.

[30] Jacomin A.C., Fauvarque M.O., Taillebourg E. (2016). A functional endosomal pathway is necessary for lysosome biogenesis in Drosophila. BMC Cell Biol..

[31] Van der Sluijs P., Hull M., Webster P., Mâle P., Goud B., Mellman I. (1992). The small GTP-binding protein rab4 controls an early sorting event on the endocytic pathway. Cell.

[32] Campa C.C., Hirsch E. (2017). Rab11 and phosphoinositides: A synergy of signal transducers in the control of vesicular trafficking. Adv. Biol. Regul..

[33] Goldenring J.R. (2015). Recycling endosomes. Curr. Opin. Cell Biol..

[34] Kaushik S., Cuervo A.M. (2012). Chaperone-mediated autophagy: A unique way to enter the lysosome world. Trends Cell Biol..

[35] Li W.W., Li J., Bao J.K. (2012). Microautophagy: Lesser-known self-eating. Cell. Mol. Life Sci..

[36] Wei Y., Liu M., Li X., Liu J., Li H. (2018). Origin of the autophagosome membrane in mammals. BioMed Res. Int..

[37] Gaudecker V. (1963). On variation in some cell organelles during formation of reserve substances in fatty bodies of *Drosophila larvae*. Z. Zellforsch. Mikrosk. Anat..

[38] Mulakkal N.C., Nagy P., Takats S., Tusco R., Juhász G., Nezis I.P. (2014). Autophagy in Drosophila: from historical studies to current knowledge. BioMed Res. Int..

[39] Lund V.K., Madsen K.L., Kjaerulff O. (2018). Drosophila Rab2 controls endosome-lysosome fusion and LAMP delivery to late endosomes. Autophagy.

[40] Sardiello M., Palmieri M., di Ronza A., Medina D.L., Valenza M., Gennarino V.A., Di Malta C., Donaudy F., Embrione V., Polishchuk R.S. (2009). A gene network regulating lysosomal biogenesis and function. Science.

[41] Yu L., McPhee C.K., Zheng L., Mardones G.A., Rong Y., Peng J., Mi N., Zhao Y., Liu Z., Wan F. (2010). Termination of autophagy and reformation of lysosomes regulated by mTOR. Nature.

[42] Chen Y., Yu L. (2018). Development of research into autophagic lysosome reformation. Mol. Cells.

[43] Rong Y., McPhee C.K., Deng S., Huang L., Chen L., Liu M., Tracy K., Baehrecke E.H., Yu L., Lenardo M.J. (2011). Spinster is required for autophagic lysosome reformation and mTOR reactivation following starvation. Proc. Natl. Acad. Sci. USA.

[44] Kilimann M.W., Oldfors A. (2015). Glycogen pathways in disease: New developments in a classical field of medical genetics. J. Inherit. Metab. Dis..

[45] Zhao H., Tang M., Liu M., Chen L. (2018). Glycophagy: An emerging target in pathology. Clin. Chim. Acta.

[46] Zirin J., Nieuwenhuis J., Perrimon N. (2013). Role of Autophagy in Glycogen Breakdown and Its Relevance to Chloroquine Myopathy. PLoS Biol..

[47] Kobayashi T., Beuchat M.H., Lindsay M., Frias S., Palmiter R.D., Sakuraba H., Parton R.G., Gruenberg J. (1999). Late endosomal membranes rich in lysobisphosphatidic acid regulate cholesterol transport. Nat. Cell Biol..

[48] Goldstein J.L., Brown M.S. (2015). A century of cholesterol and coronaries: From plaques to genes to statins. Cell.

[49] Thelen A.M., Zoncu R. (2017). Emerging roles for the lysosome in lipid metabolism. Trends Cell Biol..

[50] Liu Z., Huang X. (2013). Lipid metabolism in Drosophila: Development and disease. Acta Biochim. Biophys. Sin..

[51] Seranova E., Connolly K.J., Zatyka M., Rosenstock T.R., Barrett T., Tuxworth R.I., Sarkar S. (2017). Dysregulation of autophagy as a common mechanism in lysosomal storage diseases. Essays Biochem..

[52] Saha S., Panigrahi D.P., Patil S., Bhutia S.K. (2018). Autophagy in health and disease: A comprehensive review. Biomed. Pharmacother..

[53] Allocca M., Zola S., Bellosta P., Perveen F.K. (2018). The fruit fly, Drosophila melanogaster: Modeling of human diseases (part II). Drosophila Melanogaster Model for Recent Advances in Genetics and Therapeutics.

[54] Chintapalli V.R., Wang J., Dow J.A.T. (2007). Using FlyAtlas to identify better Drosophila melanogaster models of human disease. Nat. Genet..

[55] Pandey U.B., Nichols C.D. (2011). Human disease models in drosophila melanogaster and the role of the fly in therapeutic drug discovery. Pharmacol. Rev..

[56] Moulton M.J., Letsou A. (2016). Modeling congenital disease and inborn errors of development in Drosophila melanogaster. Dis. Model. Mech..

[57] Ryder E., Russell S. (2003). Transposable elements as tools for genomics and genetics in Drosophila. Briefings Funct. Genomics Proteomics.

[58] Bachmann A., Knust E. (2008). The Use of P-Element Transposons to Generate Transgenic Flies.

[59] Port F., Chen H.-M., Lee T., Bullock S.L. (2014). Optimized CRISPR/Cas tools for efficient germline and somatic genome engineering in Drosophila. Proc. Natl. Acad. Sci. USA.

[60] Port F., Muschalik N., Bullock S.L. (2015). Systematic evaluation of Drosophila CRISPR tools reveals safe and robust alternatives to autonomous gene drives in basic research. G3 Genes Genomes Genet..

[61] Hales K.G., Korey C.A., Larracuente A.M., Roberts D.M. (2015). Genetics on the fly: A primer on the Drosophila model system. Genetics.

[62] Gratz S.J., Rubinstein C.D., Harrison M.M., Wildonger J., O’Connor-Giles K.M. (2015). CRISPR-Cas9 genome editing in Drosophila. Curr. Protoc. Mol. Biol..

[63] Brand A.H., Perrimon N. (1993). Targeted gene expression as a means of altering cell fates and generating dominant phenotypes. Development.

[64] St Johnston D. (2002). The art and design of genetic screens: Drosophila melanogaster. Nat. Rev. Genet..

[65] Cho K.S., Bang S.M., Toh A. (2014). Lipids and lipid signaling in Drosophila models of neurodegenerative diseases. Omega-3 Faty Acids Brain Neurol. Health.

[66] Riedel F., Gillingham A.K., Rosa-Ferreira C., Galindo A., Munro S. (2016). An antibody toolkit for the study of membrane traffic in Drosophila melanogaster. Biol. Open.

[67] An P.N.T., Fukusaki E. (2018). Metabolomics: State-of-the-art technologies and applications on Drosophila melanogaster. Adv. Exp. Med. Biol..

[68] Bargal R., Avidan N., Ben-Asher E., Olender Z., Zeigler M., Frumkin A., Raas-Rothschild A., Glusman G., Lancet D., Bach G. (2000). Identification of the gene causing mucolipidosis type IV. Nat. Genet..

[69] Slaugenhaupt S.A., Acierno J.S., Helbling L.A., Bove C., Goldin E., Bach G., Schiffmann R., Gusella J.F. (1999). Mapping of the mucolipidosis type IV gene to chromosome 19p and definition of founder haplotypes. Am. J. Hum. Genet..

[70] Amir N., Ziotogora J., Bach G. (1987). Mucolipidosis type IV: Clinical spectrum and natural history. Pediatrics.

[71] Venkatachalam K., Long A.A., Elsaesser R., Nikolaeva D., Montell C. (2008). Motor deficit in a Drosophila model of mucolipidosis Type IV due to defective clearance of apoptotic cells. Cell.

[72] Wong C.-O., Li R., Montell C., Venkatachalam K. (2012). Drosophila TRPML is required for TORC1 activation. Curr. Biol..

[73] Wong C.-O., Palmieri M., Li J., Akhmedov D., Chao Y., Broadhead G.T., Zhu M.X., Berdeaux R., Collins C.A., Sardiello M. (2015). Diminished MTORC1-Dependent JNK activation underlies the neurodevelopmental defects associated with lysosomal dysfunction. Cell Rep..

[74] Onyenwoke R.U., Sexton J.Z., Yan F., Díaz M.C.H., Forsberg L.J., Major M.B., Brenman J.E. (2015). The mucolipidosis IV Ca2+ channel TRPML1 (MCOLN1) is regulated by the TOR kinase. Biochem. J..

[75] Haltia M., Goebel H.H. (2013). The neuronal ceroid-lipofuscinoses: A historical introduction. Biochim. Biophys. Acta Mol. Basis Dis..

[76] Kohlschütter A., Schulz A. (2009). Towards understanding the neuronal ceroid lipofuscinoses. Brain Dev..

[77] Glaser R.L., Hickey A.J., Chotkowski H.L., Chu-LaGraff Q. (2003). Characterization of Drosophila palmitoyl-protein thioesterase 1. Gene.

[78] Hickey A.J., Chotkowski H.L., Singh N., Ault J.G., Korey C.A., MacDonald M.E., Glaser R.L. (2006). Palmitoyl-protein thioesterase 1 deficiency in Drosophila melanogaster causes accumulation of abnormal storage material and reduced life span. Genetics.

[79] Chu-LaGraff Q., Blanchette C., O’Hern P., Denefrio C. (2010). The Batten disease Palmitoyl Protein Thioesterase 1 gene regulates neural specification and axon connectivity during Drosophila embryonic development. PLoS ONE.

[80] Munroe P.B., Mitchison H.M., O’rawe A.M., Anderson J.W., Boustany R.-M., Lerner T.J., Taschner P.E.M., De Vos N., Breuning M.H., Gardiner R.M. (1997). Spectrum of Mutations in the Batten Disease Gene, CLN3. Am. J. Hum. Genet..

[81] Tuxworth R.I., Vivancos V., O’Hare M.B., Tear G. (2009). Interactions between the juvenile Batten disease gene, CLN3, and the Notch and JNK signalling pathways. Hum. Mol. Genet..

[82] Tuxworth R.I., Chen H., Vivancos V., Carvajal N., Huang X., Tear G. (2011). The Batten disease gene CLN3 is required for the response to oxidative stress. Hum. Mol. Genet..

[83] Henderson M.X., Wirak G.S., Zhang Y.-q., Dai F., Ginsberg S.D., Dolzhanskaya N., Staropoli J.F., Nijssen P.C.G., Lam T.K.T., Roth A.F. (2016). Neuronal ceroid lipofuscinosis with DNAJC5/CSPα mutation has PPT1 pathology and exhibit aberrant protein palmitoylation. Acta Neuropathol..

[84] Nosková L., Stránecký V., Hartmannová H., Přistoupilová A., Barešová V., Ivánek R., Hlková H., Jahnová H., Van Der Zee J., Staropoli J.F. (2011). Mutations in DNAJC5, encoding cysteine-string protein alpha, cause autosomal-dominant adult-onset neuronal ceroid lipofuscinosis. Am. J. Hum. Genet..

[85] Imler E., Pyon J.S., Kindelay S., Torvund M., Zhang Y.Q., Chandra S.S., Zinsmaier K.E. (2019). A drosophila model of neuronal ceroid lipofuscinosis CLN4 reveals a hypermorphic gain of function mechanism. eLife.

[86] Myllykangas L., Tyynela J., Page-McCaw A., Rubin G.M., Haltia M.J., Feany M.B. (2005). Cathepsin D-deficient Drosophila recapitulate the key features of neuronal ceroid lipofuscinoses. Neurobiol. Dis..

[87] Kuronen M., Talvitie M., Lehesjoki A.E., Myllykangas L. (2009). Genetic modifiers of degeneration in the cathepsin D deficient Drosophila model for neuronal ceroid lipofuscinosis. Neurobiol. Dis..

[88] Fecarotta S., Tarallo A., Damiano C., Minopoli N., Parenti G. (2020). Pathogenesis of mucopolysaccharidoses, an update. Int. J. Mol. Sci..

[89] Khan S.A., Peracha H., Ballhausen D., Wiesbauer A., Rohrbach M., Gautschi M., Mason R.W., Giugliani R., Suzuki Y., Orii K.E. (2017). Epidemiology of mucopolysaccharidoses. Mol. Genet. Metab..

[90] Rathmann M., Bunge S., Beck M., Kresse H., Tylki-Szymanska A., Gal A. (1996). Mucopolysaccharidosis type II (Hunter syndrome): Mutation “hot spots” in the iduronate-2-sulfatase gene. Am. J. Hum. Genet..

[91] D’Avanzo F., Rigon L., Zanetti A., Tomanin R. (2020). Mucopolysaccharidosis type II: One hundred years of research, diagnosis, and treatment. Int. J. Mol. Sci..

[92] Young I.D., Harper P.S., Newcombe R.G., Archer I.M. (1982). A clinical and genetic study of Hunter’s syndrome. 2. Differences between the mild and severe forms. J. Med. Genet..

[93] Holmes R.S. (2017). Comparative studies of vertebrate iduronate 2-sulfatase (IDS) genes and proteins: Evolution of A mammalian X-linked gene. 3 Biotech.

[94] Rigon L., Kucharowski N., Eckardt F., Bauer R. (2020). Modeling mucopolysaccharidosis type II in the fruit fly by using the RNA interference approach. Life.

[95] Fedele A. (2015). Sanfilippo syndrome: Causes, consequences, and treatments. Appl. Clin. Genet..

[96] Neufeld E., Muenzer J. (2001). The mucopolysaccharidoses. The Online Metabolic and Molecular Bases of Inherited Disease.

[97] Webber D.L., Choo A., Hewson L.J., Trim P.J., Snel M.F., Hopwood J.J., Richards R.I., Hemsley K.M., O’Keefe L.V. (2018). Neuronal-specific impairment of heparan sulfate degradation in Drosophila reveals pathogenic mechanisms for Mucopolysaccharidosis type IIIA. Exp. Neurol..

[98] Aronovich E.L., Johnston J.M., Wang P., Giger U., Whitley C.B. (2001). Molecular basis of mucopolysaccharidosis type IIIB in Emu (Dromaius novaehollandiae): An avian model of sanfilippo syndrome type B<. Genomics.

[99] Bar S., Prasad M., Datta R. (2018). Neuromuscular degeneration and locomotor deficit in a Drosophila model of mucopolysaccharidosis VII is attenuated by treatment with resveratrol. Dis. Model. Mech..

[100] Hassan M.I., Waheed A., Grubb J.H., Klei H.E., Korolev S., Sly W.S. (2013). High Resolution crystal structure of human β-glucuronidase reveals structural basis of lysosome targeting. PLoS ONE.

[101] Grubb J.H., Vogler C., Levy B., Galvin N., Tan Y., Sly W.S. (2008). Chemically modified glucuronidase crosses blood-brain barrier and clears neuronal storage in murine mucopolysaccharidosis VII. Proc. Natl. Acad. Sci. USA.

[102] Islam M.R., Tomatsu S., Shah G.N., Grubb J.H., Jain S., Sly W.S. (1999). Active site residues of human beta-glucuronidase. Evidence for Glu(540) as the nucleophile and Glu(451) as the acid-base residue. J. Biol. Chem..

[103] Sellin J., Schulze H., Paradis M., Gosejacob D., Papan C., Shevchenko A., Psathaki O.E., Paululat A., Thielisch M., Sandhoff K. (2017). Characterization of *Drosophila Saposin-related* mutants as a model for lysosomal sphingolipid storage diseases. Dis. Model. Mech..

[104] Naureckiene S., Sleat D.E., Lackland H., Fensom A., Vanier M.T., Wattiaux R., Jadot M., Lobel P. (2000). Identification of HE1 as the second gene of Niemann-Pick C disease. Science.

[105] Carstea E.D., Morris J.A., Coleman K.G., Loftus S.K., Zhang D., Cummings C., Gu J., Rosenfeld M.A., Pavan W.J., Krizman D.B. (1997). Niemann-Pick C1 disease gene: Homology to mediators of cholesterol homeostasis. Science.

[106] Sturley S.L., Patterson M.C., Balch W., Liscum L. (2004). The pathophysiology and mechanisms of NP-C disease. Biochim. Biophys. Acta Mol. Cell Biol. Lipids.

[107] Mukherjee S., Maxfield F.R. (2004). Lipid and cholesterol trafficking in NPC. Biochim. Biophys. Acta Mol. Cell Biol. Lipids.

[108] Liscum L., Sturley S.L. (2004). Intracellular trafficking of Niemann–Pick C proteins 1 and 2: Obligate components of subcellular lipid transport. Biochim. Biophys. Acta Mol. Cell Biol. Lipids.

[109] Huang X., Suyama K., Buchanan J., Zhu A.J., Scott M.P. (2005). A Drosophila model of the Niemann-Pick type C lysosome storage disease: dnpc1a is required for molting and sterol homeostasis. Development.

[110] Huang X., Warren J.T., Buchanan J., Gilbert L.I., Scott M.P. (2007). Drosophila Niemann-Pick type C-2 genes control sterol homeostasis and steroid biosynthesis: A model of human neurodegenerative disease. Development.

[111] Fluegel M.L., Parker T.J., Pallanck L.J. (2006). Mutations of a Drosophila NPC1 gene confer sterol and ecdysone metabolic defects. Genetics.

[112] Phillips S.E., Woodruff E.A., Liang P., Patten M., Broadie K. (2008). Neuronal loss of Drosophila NPC1a causes cholesterol aggregation and age-progressive neurodegeneration. J. Neurosci..

[113] Dandana A., Ben Khelifa S., Chahed H., Miled A., Ferchichi S. (2016). Gaucher disease: Clinical, biological and therapeutic aspects. Pathobiology.

[114] Cox T.M. (2010). Gaucher disease: Clinical profile and therapeutic developments. Biologics.

[115] Robinson S.W., Herzyk P., Dow J.A.T., Leader D.P. (2013). FlyAtlas: Database of gene expression in the tissues of Drosophila melanogaster. Nucleic Acids Res..

[116] Kawasaki H., Suzuki T., Ito K., Takahara T., Goto-Inoue N., Setou M., Sakata K., Ishida N. (2017). Minos-insertion mutant of the Drosophila GBA gene homologue showed abnormal phenotypes of climbing ability, sleep and life span with accumulation of hydroxy-glucocerebroside. Gene.

[117] Davis M.Y., Trinh K., Thomas R.E., Yu S., Germanos A.A., Whitley B.N., Sardi S.P., Montine T.J., Pallanck L.J. (2016). Glucocerebrosidase Deficiency in Drosophila Results in α-Synuclein-Independent Protein Aggregation and Neurodegeneration. PLoS Genet..

[118] Maor G., Rencus-Lazar S., Filocamo M., Steller H., Segal D., Horowitz M. (2013). Unfolded protein response in Gaucher disease: From human to Drosophila. Orphanet J. Rare Dis..

[119] Cabasso O., Paul S., Dorot O., Maor G., Krivoruk O., Pasmanik-Chor M., Mirzaian M., Ferraz M., Aerts J., Horowitz M. (2019). *Drosophila melanogaster* mutated in its *GBA1b* ortholog recapitulates neuronopathic Gaucher disease. J. Clin. Med..

[120] Dasari S.K., Schejter E., Bialik S., Shkedy A., Levin-Salomon V., Levin-Zaidman S., Kimchi A. (2017). Death by over-eating: The Gaucher disease associated gene GBA1, identified in a screen for mediators of autophagic cell death, is necessary for developmental cell death in Drosophila midgut. Cell Cycle.

[121] Kinghorn K.J., Grönke S., Castillo-Quan J.I., Woodling N.S., Li L., Sirka E., Gegg M., Mills K., Hardy J., Bjedov I. (2016). A *Drosophila* model of neuronopathic Gaucher Disease demonstrates lysosomal-autophagic defects and altered mTOR signalling and is functionally rescued by rapamycin. J. Neurosci..

[122] Suzuki T., Shimoda M., Ito K., Hanai S., Aizawa H., Kato T., Kawasaki K., Yamaguchi T., Ryoo H.D., Goto-Inoue N. (2013). Expression of human Gaucher disease gene GBA generates neurodevelopmental defects and ER stress in Drosophila eye. PLoS ONE.

[123] Maor G., Cabasso O., Krivoruk O., Rodriguez J., Steller H., Segal D., Horowitz M. (2016). The contribution of mutant GBA to the development of Parkinson disease in Drosophila. Hum. Mol. Genet..

[124] Maor G., Rapaport D., Horowitz M. (2019). The effect of mutant GBA1 on accumulation and aggregation of a synuclein. Hum. Mol. Genet..

[125] Matthes F., Stroobants S., Gerlach D., Wohlenberg C., Wessig C., Fogh J., Gieselmann V., Eckhardt M., D’Hooge R., Matzner U. (2012). Efficacy of enzyme replacement therapy in an aggravated mouse model of metachromatic leukodystrophy declines with age. Hum. Mol. Genet..

[126] Van Rappard D.F., Boelens J.J., Wolf N.I. (2015). Metachromatic leukodystrophy: Disease spectrum and approaches for treatment. Best Pract. Res. Clin. Endocrinol. Metab..

[127] Lee J.S., Kanai K., Suzuki M., Kim W.S., Yoo H.S., Fu Y., Kim D.-K., Jung B.C., Choi M., Oh K.W. (2019). Arylsulfatase A, a genetic modifier of Parkinson’s disease, is an α-synuclein chaperone. Brain.

[128] Aerts J.M., Groener J.E., Kuiper S., Donker-Koopman W.E., Strijland A., Ottenhoff R., Van Roomen C., Mirzaian M., Wijburg F.A., Linthorst G.E. (2008). Elevated globotriaosylsphingosine is a hallmark of Fabry disease. Proc. Natl. Acad. Sci. USA.

[129] Schiffmann R., Moore D.F., Mehta A., Beck M., Sunder-Plassmann G. (2006). Neurological Manifestations of Fabry Disease. Fabry Disease: Perspectives from 5 Years of FOS.

[130] McCafferty E.H., Scott L.J. (2019). Migalastat: A Review in Fabry Disease. Drugs.

[131] Kalliokoski R.J., Kalliokoski K.K., Penttinen M., Kantola I., Leino A., Viikari J.S., Simell O., Nuutila P., Raitakari O.T. (2006). Structural and functional changes in peripheral vasculature of Fabry patients. J. Inherit. Metab. Dis..

[132] Barbey F., Brakch N., Linhart A., Jeanrenaud X., Palecek T., Bultas J., Burnier M., Hayoz D. (2007). Increased carotid intima-media thickness in the absence of atherosclerotic plaques in an adult population with Fabry disease. Acta Paediatr..

[133] Braunstein H., Papazian M., Maor G., Lukas J., Rolfs A., Horowitz M. (2020). Misfolding of lysosomal α-galactosidase a in a fly model and its alleviation by the pharmacological chaperone migalastat. Int. J. Mol. Sci..

[134] Burkhardt J.K., Hüttler S., Klein A., Möbius W., Habermann A., Griffiths G., Sandhoff K. (1997). Accumulation of sphingolipids in SAP-precursor (prosaposin)-deficient fibroblasts occurs as intralysosomal membrane structures and can be completely reversed by treatment with human SAP-precursor. Eur. J. Cell Biol..

[135] Hindle S.J., Hebbar S., Schwudke D., Elliott C.J.H., Sweeney S.T. (2017). A saposin deficiency model in Drosophila: Lysosomal storage, progressive neurodegeneration and sensory physiological decline. Neurobiol. Dis..

[136] Huizing M., Helip-Wooley A., Westbroek W., Gunay-Aygun M., Gahl W.A. (2008). Disorders of lysosome-related organelle biogenesis: Clinical and molecular genetics. Annu. Rev. Genomics Hum. Genet..

[137] Hebbar S., Khandelwal A., Jayashree R., Hindle S.J., Chiang Y.N., Yew J.Y., Sweeney S.T., Schwudke D. (2017). Lipid metabolic perturbation is an early-onset phenotype in adult *spinster* mutants: A *Drosophila* model for lysosomal storage disorders. Mol. Biol. Cell.

[138] Sweeney S.T., Davis G.W. (2002). Unrestricted Synaptic Growth in spinster—a late endosomal protein implicated in TGF-β-Mediated synaptic growth regulation. Neuron.

[139] Dermaut B., Norga K.K., Kania A., Verstreken P., Pan H., Zhou Y., Callaerts P., Bellen H.J. (2005). Aberrant lysosomal carbohydrate storage accompanies endocytic defects and neurodegeneration in Drosophila benchwarmer. J. Cell Biol..

[140] Fernández-Hernández I., Scheenaard E., Pollarolo G., Gonzalez C. (2016). The translational relevance of Drosophila in drug discovery. EMBO Rep..

[141] Willoughby L.F., Schlosser T., Manning S.A., Parisot J.P., Street I.P., Richardson H.E., Humbert P.O., Brumby A.M. (2013). An in vivo large-scale chemical screening platform using Drosophila for anti-cancer drug discovery. Dis. Model. Mech..

[142] Limmer S., Weiler A., Volkenhoff A., Babatz F., Klämbt C. (2014). The Drosophila blood-brain barrier: Development and function of a glial endothelium. Front. Neurosci..

[143] Daneman R., Barres B.A. (2005). The blood-brain barrier-Lessons from moody flies. Cell.

[144] Schulman V.K., Folker E.S., Baylies M.K. (2013). A method for reversible drug delivery to internal tissues of Drosophila embryos. Fly.

[145] Fantin M., Garelli F., Napoli B., Forgiarini A., Gumeni S., De Martin S., Montopoli M., Vantaggiato C., Orso G. (2019). Flavonoids regulate lipid droplets biogenesis in *Drosophila melanogaster*. Nat. Prod. Commun..

[146] Napoli B., Gumeni S., Forgiarini A., Fantin M., De Filippis C., Panzeri E., Vantaggiato C., Orso G. (2019). Naringenin ameliorates Drosophila ReepA hereditary spastic paraplegia-linked phenotypes. Front. Neurosci..

[147] Forgiarini A., Wang Z., D’Amore C., Jay-Smith M., Li F.F., Hopkins B., Brimble M.A., Pagetta A., Bersani S., De Martin S. (2019). Live applications of norbormide-based fluorescent probes in Drosophila melanogaster. PLoS ONE.

[148] Zabihihesari A., Hilliker A.J., Rezai P. (2020). Localized microinjection of intact *Drosophila melanogaster* larva to investigate the effect of serotonin on heart rate. Lab Chip.

[149] Nichols C.D., Ronesi J., Pratt W., Sanders-Bush E. (2002). Hallucinogens and Drosophila: Linking serotonin receptor activation to behavior. Neuroscience.

[150] Turin L., Skoulakis E.M.C., Horsfield A.P. (2014). Electron spin changes during general anesthesia in Drosophila. Proc. Natl. Acad. Sci. USA.

[151] Zalucki O.H., Menon H., Kottler B., Faville R., Day R., Bademosi A.T., Lavidis N., Karunanithi S., Van Swinderen B. (2015). Syntaxin1A-mediated Resistance and Hypersensitivity to Isoflurane in Drosophila melanogaster. Anesthesiology.

[152] Moore M.S., DeZazzo J., Luk A.Y., Tully T., Singh C.M., Heberlein U. (1998). Ethanol intoxication in Drosophila: Genetic and pharmacological evidence for regulation by the cAMP signaling pathway. Cell.

[153] McClung C., Hirsh J. (1998). Stereotypic behavioral responses to free-base cocaine and the development of behavioral sensitization in drosophila. Curr. Biol..

[154] Poudel S., Kim Y., Kwak J.S., Jeong S., Lee Y. (2017). Gustatory receptor 22e is essential for sensing chloroquine and strychnine in Drosophila melanogaster. Insect Biochem. Mol. Biol..

[155] Charlu S., Wisotsky Z., Medina A., Dahanukar A. (2013). Acid sensing by sweet and bitter taste neurons in Drosophila melanogaster. Nat. Commun..

[156] Gasque G., Conway S., Huang J., Rao Y., Vosshall L.B. (2013). Small molecule drug screening in Drosophila identifies the 5HT2A receptor as a feeding modulation target. Sci. Rep..

[157] Ja W.W., Carvalho G.B., Mak E.M., De La Rosa N.N., Fang A.Y., Liong J.C., Brummel T., Benzer S. (2007). Prandiology of Drosophila and the CAFE assay. Proc. Natl. Acad. Sci. USA.

[158] Deshpande S.A., Carvalho G.B., Amador A., Phillips A.M., Hoxha S., Lizotte K.J., Ja W.W. (2014). Quantifying Drosophila food intake: Comparative analysis of current methodology. Nat. Methods.

[159] Shell B.C., Schmitt R.E., Lee K.M., Johnson J.C., Chung B.Y., Pletcher S.D., Grotewiel M. (2018). Measurement of solid food intake in Drosophila via consumption-excretion of a dye tracer. Sci. Rep..

[160] Kuklinski N.J., Berglund E.C., Ewing A.G. (2010). Micellar capillary electrophoresis-Electrochemical detection of neurochemicals from Drosophila. J. Sep. Sci..

[161] Levario T.J., Zhao C., Rouse T., Shvartsman S.Y., Lu H. (2016). An integrated platform for large-scale data collection and precise perturbation of live Drosophila embryos. Sci. Rep..

[162] Ali S.N., Dayarathna T.K., Ali A.N., Osumah T., Ahmed M., Cooper T.T., Power N.E., Zhang D., Kim D., Kim R. (2018). Drosophila melanogaster as a function-based high-throughput screening model for antinephrolithiasis agents in kidney stone patients. DMM Dis. Model. Mech..

[163] Filošević A., Al-samarai S., Andretić Waldowski R. (2018). High Throughput measurement of locomotor sensitization to volatilized cocaine in Drosophila melanogaster. Front. Mol. Neurosci..

[164] Giacomotto J., Ségalat L. (2010). High-throughput screening and small animal models, where are we?. Br. J. Pharmacol..

[165] Yadav A.K., Srikrishna S., Gupta S.C. (2016). Cancer drug development using Drosophila as an in vivo Tool: From bedside to bench and back. Trends Pharmacol. Sci..

[166] Castillo-Quan J.I., Tain L.S., Kinghorn K.J., Li L., Grönke S., Hinze Y., Blackwell T.K., Bjedov I., Partridge L. (2019). A triple drug combination targeting components of the nutrient-sensing network maximizes longevity. Proc. Natl. Acad. Sci. USA.

[167] Segarra M., Aburto M.R., Acker-Palmer A. (2021). Blood–brain barrier dynamics to maintain brain homeostasis. Trends Neurosci..

[168] Benz F., Liebner S. (2020). Structure and Function of the Blood–Brain Barrier (BBB).

[169] O’Brown N.M., Pfau S.J., Gu C. (2018). Bridging barriers: A comparative look at the blood-brain barrier across organisms. Genes Dev..

[170] Rotstein B., Paululat A. (2016). On the morphology of the Drosophila Heart. J. Cardiovasc. Dev. Dis..

[171] Stork T., Engelen D., Krudewig A., Silies M., Bainton R.J., Klämbt C. (2008). Organization and function of the blood-brain barrier in Drosophila. J. Neurosci..

[172] Rouka E., Gourgoulianni N., Lüpold S., Hatzoglou C., Gourgoulianis K., Blanckenhorn W.U., Zarogiannis S.G. (2021). The Drosophila septate junctions beyond barrier function: Review of the literature, prediction of human orthologs of the SJ-related proteins and identification of protein domain families. Acta Physiol..

[173] Gupta S.K., Singh P., Ali V., Verma M. (2020). Role of membrane-embedded drug efflux ABC transporters in the cancer chemotherapy. Oncol. Rev..

[174] Sun H., Buchon N., Scott J.G. (2017). Mdr65 decreases toxicity of multiple insecticides in Drosophila melanogaster. Insect Biochem. Mol. Biol..

[175] DeSalvo M.K., Hindle S.J., Rusan Z.M., Orng S., Eddison M., Halliwill K., Bainton R.J. (2014). The Drosophila surface glia transcriptome: Evolutionary conserved blood-brain barrier processes. Front. Neurosci..

[176] Pellerin L., Magistretti P.J. (2011). Sweet sixteen for ANLS. J. Cereb. Blood Flow Metab..

[177] Fünfschilling U., Supplie L.M., Mahad D., Boretius S., Saab A.S., Edgar J., Brinkmann B.G., Kassmann C.M., Tzvetanova I.D., Möbius W. (2012). Glycolytic oligodendrocytes maintain myelin and long-term axonal integrity. Nature.

[178] Holcroft C.E., Jackson W.D., Lin W.-H., Bassiri K., Baines R.A., Phelan P. (2013). Innexins Ogre and Inx2 are required in glial cells for normal postembryonic development of the Drosophila central nervous system. J. Cell Sci..

[179] Spéder P., Brand A.H. (2014). Gap Junction proteins in the blood-brain barrier control nutrient-dependent reactivation of Drosophila neural stem cells. Dev. Cell.

[180] Liu L., Zhang K., Sandoval H., Yamamoto S., Jaiswal M., Sanz E., Li Z., Hui J., Graham B.H., Quintana A. (2015). Glial lipid droplets and ROS induced by mitochondrial defects promote neurodegeneration. Cell.

[181] Liu L., MacKenzie K.R., Putluri N., Maletić-Savatić M., Bellen H.J. (2017). The glia-neuron lactate shuttle and elevated ROS promote lipid synthesis in neurons and lipid droplet accumulation in glia via APOE/D. Cell Metab..

[182] Bliss T.M., Ip M., Cheng E., Minami M., Pellerin L., Magistretti P., Sapolsky R.M. (2004). Dual-gene, dual-cell type therapy against an excitotoxic insult by bolstering neuroenergetics. J. Neurosci..

[183] Berthet C., Lei H., Thevenet J., Gruetter R., Magistretti P.J., Hirt L. (2009). Neuroprotective role of lactate after cerebral ischemia. J. Cereb. Blood Flow Metab..

[184] Noe C.R., Noe-Letschnig M., Handschuh P., Noe C.A., Lanzenberger R. (2020). Dysfunction of the blood-brain barrier—A key step in neurodegeneration and dementia. Front. Aging Neurosci..

[185] Shigemoto-Mogami Y., Hoshikawa K., Sato K. (2018). Activated microglia disrupt the blood-brain barrier and induce chemokines and cytokines in a rat in vitro model. Front. Cell. Neurosci..

[186] Kwon H.S., Koh S.H. (2020). Neuroinflammation in neurodegenerative disorders: The roles of microglia and astrocytes. Transl. Neurodegener..

[187] Doherty J., Logan M.A., Taşdemir Ö.E., Freeman M.R. (2009). Ensheathing glia function as phagocytes in the adult Drosophila brain. J. Neurosci..

[188] Hakim-Mishnaevski K., Flint-Brodsly N., Shklyar B., Levy-Adam F., Kurant E. (2019). Glial phagocytic receptors promote neuronal loss in adult Drosophila brain. Cell Rep..

[189] Obermeier B., Daneman R., Ransohoff R.M. (2013). Development, maintenance and disruption of the blood-brain barrier. Nat. Med..

[190] Cuddapah V.A., Zhang S.L., Sehgal A. (2019). Regulation of the blood–brain barrier by circadian rhythms and sleep. Trends Neurosci..

[191] Zhang S.L., Yue Z., Arnold D.M., Artiushin G., Sehgal A. (2018). A Circadian clock in the blood-brain barrier regulates xenobiotic efflux. Cell.

[192] Sarantseva S.V., Bolshakova O.I., Timoshenko S.I., Kolobov A.A., Schwarzman A.L. (2011). Dendrimer D5 is a vector for peptide transport to brain cells. Bull. Exp. Biol. Med..

